# The evolution of multiple active site configurations in a designed enzyme

**DOI:** 10.1038/s41467-018-06305-y

**Published:** 2018-09-25

**Authors:** Nan-Sook Hong, Dušan Petrović, Richmond Lee, Ganna Gryn’ova, Miha Purg, Jake Saunders, Paul Bauer, Paul D. Carr, Ching-Yeh Lin, Peter D. Mabbitt, William Zhang, Timothy Altamore, Chris Easton, Michelle L. Coote, Shina C. L. Kamerlin, Colin J. Jackson

**Affiliations:** 1 Research School of Chemistry, Australian National University, Canberra, ACT 2601 Australia; 2 Department of Chemistry, BMC, Uppsala University, Box 576, 751 23 Uppsala, Sweden; 3 Institut des Sciences et Ingénierie Chimiques, École Polytechnique Fédérale de Lausanne, 1015 Lausanne, Switzerland

## Abstract

Developments in computational chemistry, bioinformatics, and laboratory evolution have facilitated the de novo design and catalytic optimization of enzymes. Besides creating useful catalysts, the generation and iterative improvement of designed enzymes can provide valuable insight into the interplay between the many phenomena that have been suggested to contribute to catalysis. In this work, we follow changes in conformational sampling, electrostatic preorganization, and quantum tunneling along the evolutionary trajectory of a designed Kemp eliminase. We observe that in the Kemp Eliminase KE07, instability of the designed active site leads to the emergence of two additional active site configurations. Evolutionary conformational selection then gradually stabilizes the most efficient configuration, leading to an improved enzyme. This work exemplifies the link between conformational plasticity and evolvability and demonstrates that residues remote from the active sites of enzymes play crucial roles in controlling and shaping the active site for efficient catalysis.

## Abstract (web summary)

Generation and iterative optimization of designed enzymes can provide valuable insights for a more efficient catalysis. Here the authors have followed the iterative improvement of a designed Kemp eliminase and show that remote point mutations could remodel the designed active site via substantial conformational reorganization.

## Introduction

Efficient de novo computational enzyme design has been a long-held goal of protein engineers and would allow the catalytic power of enzymes to be directed towards a range of industrially and medically important chemical reactions. Studies have demonstrated that although de novo design is possible, the imperfect designs often require optimization through laboratory evolution^1,2^. Our ability to design enzymes rests upon our fundamental understanding of enzyme catalysis, yet the biophysical and chemical basis for their catalytic efficiency remains a topic of debate^3–5^. There is evidence for contributions to catalysis from electrostatic transition state (TS) stabilization, conformational changes, and quantum tunneling^6–8^. Conformational sampling has been shown to allow enzymes to adopt specific configurations that are suited to different steps in their catalytic cycle and recent work has shown how remote mutations can alter the conformational landscape to increase sampling of certain conformational substates^7,9^. Vibrational motions have also been suggested to contribute to the chemical step in catalysis by altering the probability of transmission through the TS barrier in some enzymes by quantum mechanical hydrogen tunneling^8,10^.

**Fig. 1:**
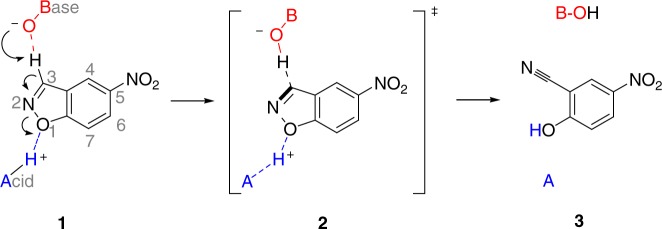
Reaction scheme for the Kemp elimination of 5-nitrobenzisoxazole. The nucleophilic oxygen atom of the base (B) donates electrons to the electrophilic 3′-H of 5-nitrobenzisoxazole and the electronegative oxygen atom of the isoxazole group forms a hydrogen bond with an acid (A) (**1**), forming a transition state in which the C−H and N−O bonds are weakened (**2**). The removal of the 3′-H from the substrate leads to an anionic phenoxide intermediate, which is then protonated, forming the final product (**3**)

Kemp elimination (proton elimination from 5-nitrobenzisoxazole; Fig. 1) has been extensively used as a model system in enzyme design owing to the simplicity of the base-catalyzed ring opening reaction^11^ and the absence of natural Kemp eliminases^1^, although some enzymes have been shown to catalyze Kemp elimination promiscuously^12,13^. Computational design of KE07 involved construction of a theozyme to catalyze the chemical reaction, which was then grafted into the scaffold of imidazole glycerol phosphate synthase (HisF) from *Thermotoga maritima*^1^. Catalytically essential residues from the initial design include a base (Glu101) that facilitates C−H bond cleavage, an H-bond donor (Lys222) to stabilize the phenoxide intermediate, and a π-stacking residue (Trp50), which was designed to stabilize the transition state and favor substrate binding through interactions with the aromatic ring of the substrate. This initial KE07 design (Round 1; R1) catalyzes the cleavage of 5-nitrobenzisoxazole (**1**), with 10^3^-fold rate acceleration over the noncatalyzed reaction and a turnover rate (*k*_cat_) of 0.018 s^−1^. Seven generations of directed evolution then enhanced this turnover-rate over 100-fold^1^.

Although the improvements to KE07 have been partially rationalized through experimental and computational characterization of the mutant proteins^14–17^, accounting for the effects of remote mutations in later rounds has been challenging. KE07 is not the most efficient of the several Kemp eliminases now designed^18–20^, but in the context of understanding how enzyme activity can be gradually improved through stepwise mutations, its low efficiency makes it an ideal model system to study the mechanisms by which evolution or engineering can improve an inefficient starting point.

In this study we use a combination of protein crystallography, enzyme kinetics, and computational approaches to investigate the structure, function, and dynamics of a series of improved variants of the KE07 series. By soaking crystals of various KE07 variants with substrate, we capture the enzymes with a series of different active site configurations. Using molecular dynamics simulations to investigate the sampling of the different conformational substates, we show that the evolutionary improvement of KE07 involves conformational selection of an alternative, nondesigned, active site configuration.

## Results

### Computational design and initial catalytic improvement in rounds 1–4

To investigate the progressive increase in catalytic activity, we determined Arrhenius parameters for several variants, including the activation energy (*E*_a_; associated with the enthalpy of the reaction) and pre-exponential factor (*A*; associated with frequency of collisions between molecules, or entropy) (Table 1). From R1 to R4 we observe a significant reduction in the *E*_a_, from 10.8 kcal mol^−1^ (R1) to 5.6 kcal mol^−1^ (R4), indicating that the Ile7Asp, Lys146Glu, Gly202Arg, and Asn224Asp mutations substantially improve the enzyme’s ability to catalyze the reaction, primarily through enthalpic effects such as an increase in basicity of the catalytic group or improved TS stabilization. However, the significant reduction in the activation energy from R1 to R4 was offset by a less favorable pre-exponential factor (containing the entropic component, or collision frequency)^21^ (Table 1).

**Table 1:** Arrhenius parameters

KE07 variant	^a^*k*_H_ (s^−^^1^)	^a^*K*_M_ (H) (mM)	^a^*k*_cat_/*K*_M_ (H) (s^−^^1^∙M^−1^)	*E*_a(H)_^b^	*E*_a(D)_	*E*_a(D)_−*E*_a(H)_	ln*A*_H_	ln*A*_D_	*A*_H_/*A*_D_	*k*_H_ */k*_D_
^c^B	3.25E-05 (1E-06)	—	—	14.5 (0.8)	16.7 (0.7)	2.3 (1.1)	13.7 (1.4)	16.0 (1.1)	0.107 (0.01)	4.6 (0.1)
R1	0.02 (0.0008)	0.96 (0.06)	24.5 (1.8)	10.8 (1.1)	15.1 (1.0)	4.3 (1.5)	14.0 (1.8)	19.6 (1.7)	0.004 (0.0006)	4.9 (0.2)
R4	0.96 (0.09)	1.10 (0.17)	877 (159)	5.6 (0.8)	9.8 (0.8)	4.2 (1.2)	9.1 (1.3)	14.4 (1.4)	0.005 (0.0009)	5.6 (0.2)
R5	1.43 (0.06)	0.53 (0.05)	2700 (257)	7.7 (0.8)	9.0 (0.7)	1.4 (1.1)	12.9 (1.3)	13.0 (1.2)	0.875 (0.1211)	8.6 (0.2)
R6	1.56 (0.07)	0.50 (0.05)	3100 (332)	9.5 (0.5)	11.7 (0.5)	2.2 (0.7)	16.1 (0.8)	17.8 (0.8)	0.181 (0.0126)	6.5 (0.1)
R7	2.51 (0.14)	0.58 (0.07)	4310 (568)	7.1 (0.7)	11.0 (0.9)	4.0 (1.1)	12.5 (1.1)	17.1 (1.5)	0.009 (0.0011)	7.0 (0.2)
R7-2	3.83 (0.17)	0.55 (0.05)	6970 (669)	6.9 (0.4)	11.3 (0.5)	4.4 (0.6)	12.7 (0.7)	18.1 (0.8)	0.005 (0.0003)	7.3 (0.1)

^a^Values, at 303 K, were from Michaelis–Menten saturation curves for the enzyme reaction using a substrate concentration range of 0.1–1.2 mM of 5-nitrobenzisoxazole from two independent experiments

^b^*E*_a_ is in kcal mol^−1^. *E*_a_, ln *A* and *k*_H_/*k*_D_ values were calculated from the Arrhenius equation from rate constants measured at a range of temperatures (283–323 K) at pH 7.25 from three independent experiments. Arrhenius equations are shown in the Methods section and Arrhenius plot is shown in Supplementary Fig. 11. Propagated standard errors in the fitted parameters are in parentheses

^c^B stands for the nonenzymatic reaction rate in buffer

**Fig. 2:**
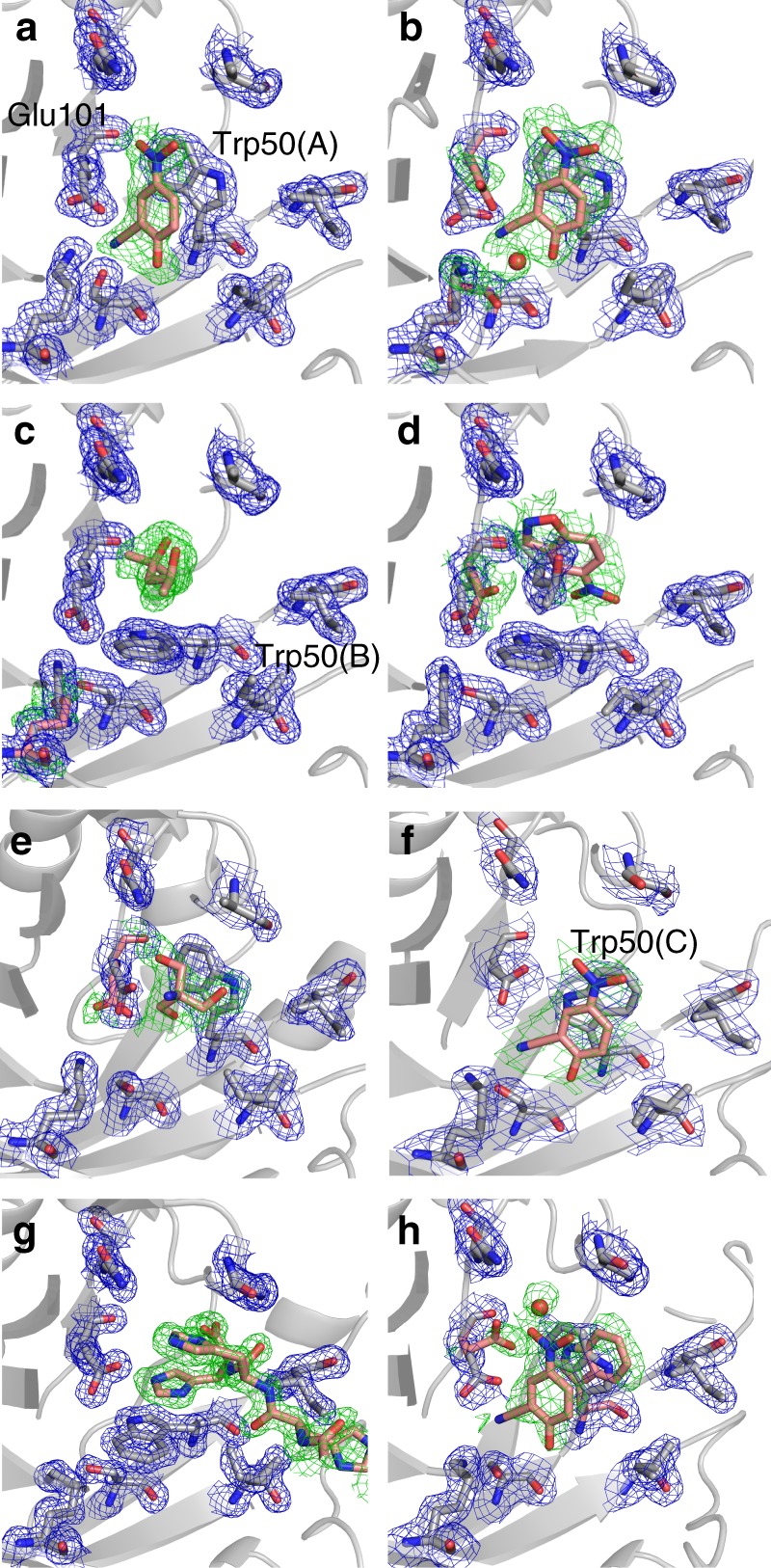
Crystal structures of different KE07 variants with and without ligand. **a** R1 in configuration A with bound product after substrate soaking. **b** R5 in configuration A with bound product after substrate soaking. **c** R5 in configuration B with bound MPD after cryoprotection. **d** R5 in configuration B with both MPD and substrate bound after substrate soaking. **e** R7 in configuration A with bound Bis-Tris molecule after cryoprotection. **f** R7 in configuration C with bound product after substrate soaking. **g** R7-2 in configuration B with bound hexahistidine tag from an adjacent protein chain after cryoprotection. **h** R7-2 in configuration C with bound product after substrate soaking, minor occupancy of configuration A is also observed. Ligands and alternative conformations of residues are shown as pink sticks. The m*F*_o_-D*F*_c_ omit maps are shown as green meshes and contoured at 3.0*σ*. The 2m*F*_o_-D*F*_c_ maps are shown as blue meshes and contoured at 1.0*σ*. PDB ID of the structures: **a** (5D2W), **b** (6DKV), **c** (6C7M), **d** (6DNJ), **e** (6CAI), **f** (6DC1), **g** (5D38), **h** (6CT3)

Analysis of crystal structures of KE07 (Supplementary Data 1), with and without bound ligands, reveals how the mutations have increased the basicity of Glu101 and enhanced TS stabilization by changing the electrostatic character of the active site. The structure of KE07 R1 was soaked with substrate, allowing us to obtain a complex with the product of the Kemp elimination (through in crystallo substrate turnover). At higher pH values (8.50 vs. 7.25) we observed mixed occupancy between the product and the histidine tag of a neighboring protein molecule in the crystal lattice within the active site, presumably because deprotonation of the structurally analogous imidazole groups within the histidine tag at basic pH increases its affinity for the active site (Supplementary Fig. 1). At pH 7.25 we observe almost full occupancy of the product (Fig. 2a). Compared with the apo-enzyme, presence of the ligand induces Lys222 to move from the salt bridge it forms with Glu101 to coordinate the oxyanion of the product (**2**). It has previously been shown that once the trajectory had reached R4, a salt bridge between Lys222 and the Ile7Asp mutation replaces the Glu101 to Lys222 salt bridge^14^ (Supplementary Fig. 2), thereby increasing the basicity of Glu101. Indeed, PROPKA^22^ suggests the p*K*_a_ of Glu101 increases from 4.49 (R1) to 6.04 (average of six chains in R4; 3IIO (3IIO)).

**Fig. 3:**
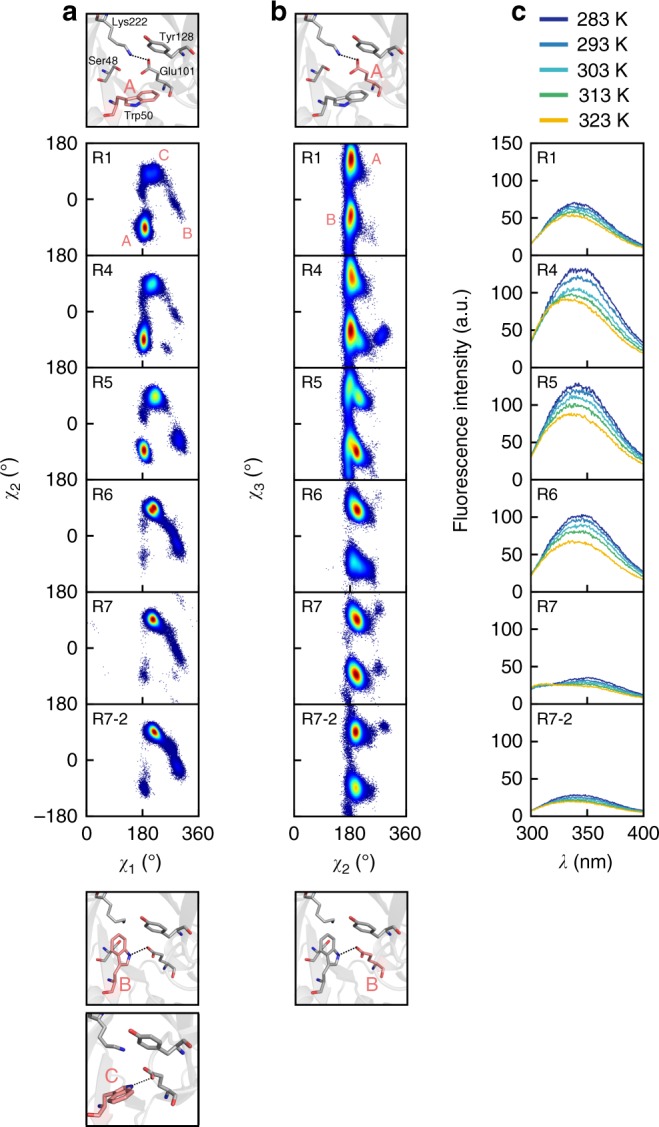
Molecular dynamics and tryptophan fluorescence of KE07 variants. Conformational sampling of **a** Trp50 and **b** Glu101 during KE07 evolution in Hamiltonian replica exchange molecular dynamics (HREX-MD) simulations and **c** tryptophan fluorescence spectra over a temperature range 283−323 K. The dihedral angle distributions of Trp50 (**a**) and Glu101 (**b**) of the KE07 variants (R1 to R7-2) are shown. Initial Trp50 conformations, together with the backbone RMSD profiles, are shown in Supplementary Fig. 7. The atoms used for dihedral angle analysis are χ1: N:CA:CB:CG, χ2: CA:CB:CG:CD1 of Trp50 (**a**). χ2: CA:CB:CG:CD, χ3: CB:CG:CD:OE1 of Glu101. The protein structures indicate the labeling used for the different Trp50 and Glu101 conformers sampled by KE07 variants. **c** Fluorescence emission spectra (excitation at 280 nm) were measured at 283−323 K (temperatures for each spectra are indicated with different colors). The fluorescence spectra of Trp50Ala mutants (R1_Trp50Ala and R7-2_Trp50Ala) are shown in Supplementary Fig. 3

To test whether these in crystallo and in silico observations were consistent with the enzyme in vitro, we used fluorescence spectroscopy. Trp50 in the active site of KE07 is a convenient spectroscopic handle to interrogate the active site environment (a second tryptophan residue, Trp156, is invariant throughout evolution; Supplementary Fig. 3). A Trp50Ala mutation was made in KE07 R1 to assess the contribution of Trp50 to the fluorescence; the crystal structures showed no major changes to the active site nor overall fold (Supplementary Fig. 4). This showed that the overall fluorescence of the R1 Trp50Ala mutant was slightly lower than R1, which is consistent with some quenching of the fluorescence of Trp156 by Trp50, similar to what has been reported previously^23^. It also revealed that the temperature dependence of KE07 R1 fluorescence is due to Trp50, with the fluorescence of the Trp50Ala variant showing almost no temperature dependence (Supplementary Fig. 3C). We observe that the Trp fluorescence of KE07 increases substantially from R1 to R4, which can reasonably be attributed to the changing environment of Trp50, given all of these mutations are in close vicinity (Fig. 3, Supplementary Fig. 2, Supplementary Table 1). The increase in fluorescence is consistent with increased negative charge on Glu101, as it has been shown that negative charge (i.e. on Glu101, which is sandwiched between Trp50 and Tyr128; Supplementary Fig. 2) will increase the quantum yield from tryptophan if it is closer to an acceptor (Tyr128) than the indole ring (Trp50)^24^. The temperature dependence of the fluorescence also increases from R1 to R4, consistent with the environment around Trp50 becoming more polarized (Supplementary Fig. 2). Thus, the solution spectroscopic behavior of KE07 R1 and R4 is consistent with the structural observations.

The loss of the salt-bridge to Lys222 reduces any H-bonding conformational constraint on the catalytic general base Glu101, which suggests that increased disorder of the catalytic residue is a plausible cause of the less favorable pre-exponential factor in R4. We examined the conformational sampling of Glu101 in R1 and R4 via Hamiltonian replica exchange molecular dynamics (HREX-MD) simulations (Fig. 3). The simulations show that the disorder of Glu101 does indeed increase from R1 to R4 (Fig. 3b). Therefore, although the electrostatic environment of KE07 may have changed to lower the activation energy, the probability of the enzyme substrate complex colliding in the correct orientation is also reduced, owing to the increased disorder of the catalytic group (Glu101), resulting in a catalytic trade-off between *E*_a_ and *A* (Table 1).

### Catalytic improvement through conformational selection

In contrast to the improvement in turnover rate across the first four rounds of mutagenesis, which was unambiguously the result of reduced activation energy due to changes to the local electrostatics of the active site via mutations within and near the active site, the catalytic efficiency of R5 and R6 is improved by more favorable collision frequency (the activation energy actually increases) (Table 1). Thus, the mechanism by which the remote mutations that appear in R5 and R6 increase activity is qualitatively different. This is mirrored in their location within the protein; in contrast to the initial mutations that affect the active site directly, the mutations in R5 (Val12Met) and R6 (Lys146Thr) are remote from the active site (Supplementary Table 2). The mutations in R7 and R7-2 (Phe77Ile, Phe229Ser, Ile102Phe) result in further optimization of activation energy.

To understand the effect of these remote mutations on catalysis, crystals of KE07 R5 and R6 were soaked with substrate before flash-cooling to 100 K and data collection (Supplementary Data 1). Soaking of the genuine substrate was preferred to cocrystallization with an analog, to eliminate any possible artifacts that changes to the chemistry of the substrate could produce. We tested a variety of cryobuffers and crystallization conditions. When glycerol was used as the soaking buffer in R5, we captured the active site in the designed conformation, into which we were able to soak substrate to capture the product bound state (Fig. 2b). In contrast, when MPD was used as the cryobuffer, we captured the active site in a different configuration, with the tryptophan residue in the active site rotating ~100° (Fig. 2c). This configuration has been observed previously in R7, although it was thought to be an artifact due to the presence of the histidine tag from a neighboring protein molecule^14^. We were able to capture the slow substrate monodeuterated (at the 3′-hydrogen) 5-nitrobenzisoxazole bound to this configuration (hereafter denoted configuration B) in a catalytically competent orientation, with C3 pointed towards the general base Glu101 (which undergoes rearrangement upon ligand binding to orient towards the substrate). These results demonstrate that substantial conformational change occurs from R5, with both configurations capable of binding substrate in a catalytically competent fashion. We also solved a higher resolution structure (1.61 Å) of R6, in which the active site of both monomers in the asymmetric unit were observed to be fully in configuration B, with the imidazole ring (an analog of the benzisoxale substrate) of the histidine tag of a neighboring KE07 molecule in the crystal lattice bound to the active site (Supplementary Fig. 1). For R7 and R7-2, we were able to capture the active site in configuration A when glycerol/Bis-Tris was used as cryoprotectant (Fig. 2e), and configuration B with the neighboring hexahistidine tag bound and MPD used as cryoprotectant (Fig. 2g). However, the most striking result came from a crystal structure with 12 different KE07 molecules within the asymmetric unit‚ into which substrate was soaked. In this structure, we observe both configuration A (three subunits) and B (one subunit) (Supplementary Fig. 5), as well as two additional configurations (hereafter denoted configurations C and D). In configuration C, Trp50 is rotated back to a similar position as the designed conformation, except it is rotated such that the NH of the indole ring can hydrogen bond to the catalytic Glu101. The four subunits in configuration C all had product bound (Fig. 2f; Supplementary Fig. 5), and were the only chains where product was observed. Four subunits were observed to adopt configuration D, in which Trp50 was disordered in-between the well-defined configurations, supporting the idea that these conformational substates exist in equilibrium (Supplementary Fig. 5). Finally, crystals of R7-2 with the hexahistidine tag crystallized in configuration B with the hexahistidine tag in the active site (even when soaked with substrate) (Fig. 2g). However, when we removed the hexahistidine tag, we captured a high-resolution structure in configuration C with product bound (configuration A was present in low occupancy; Fig. 2h). Altogether, we have characterized the emergence of two additional catalytically competent active site configurations in KE07 that were not part of the original design, have used substrate soaking and flash cooling to demonstrate that all three can bind substrate in catalytically competent orientations, and show that within the population of enzyme molecules within a single crystal, all three (and an intermediate state) can be sampled. However, in later generations (R7 and R7-2) evidence for turnover was only observed with configuration C.

The structural basis for the dramatic reorganization of the active site of KE07 is coincident with the introduction of remote mutations and appears to involve differential stabilization of the three conformational substates. For example, Ile102Phe results in phenylalanine filling a hydrophobic cavity and a small adjustment of the main chain, which increases the distance between Glu101 and Trp50, allowing sampling of the Trp50 rotamer, which is then stabilized by an H-bond to Glu101, thereby stabilizing this alternative configuration (Supplementary Fig. 6). The Val12Met and Phe77Ile mutations, also in the second shell, cause changes to internal cavities that cause a slight rotation in the backbone at Trp50 that also favors the alternative configuration (Supplementary Fig. 6). From configuration B, the enzyme can more easily access configuration C as the indole ring has already rotated at this point.

The crystallography provides valuable snap-shots of different configurations that the active site can adopt, and using different buffers, we were able to selectively stabilize certain configurations, but the crystallography does not tell us much regarding their relative populations in solution (other than that they can all be sampled). To the solution sampling of these states, we used HREX-MD simulations, which are among the most comprehensive computational methods to investigate conformational sampling^25^. These results were consistent with the crystallography and kinetic analysis: whereas in R1 (original design), configuration A was the dominant substate that was sampled, by R5 we observe increased sampling of configurations B and C (Fig. 3; Supplementary Fig. 7). By R7 and R7-2, configuration C (which was the only state we saw associated with product in the crystal structures) becomes the dominant substate sampled, with configurations A and B sampled only rarely. These simulations also reveal that the mobility of Glu101 was reduced as a result of the hydrogen bond formed to the indole nitrogen of Trp50 in R5; this could account for the improved pre-exponential factor from R5 onwards (Figs. 2,3).

We again used tryptophan fluorescence to complement the crystallographic and computational analyses. In contrast to the increase in Trp50 fluorescence intensity over the first half of the evolutionary trajectory owing to the increased negative charge of Glu101, the fluorescence intensity decreases to below the level of R1 from R4 to R7-2 (Fig. 3; Supplementary Table 1). This marked reduction in fluorescence intensity is coincident with the introduction of remote mutations (generally conservative in terms of charge) that are unlikely to directly affect the local electrostatic environment of Trp50. The loss of fluorescence is consistent with the alternative active site configurations (B and C), in which Trp50 fluorescence is quenched: either via H-bonds with Glu101 in configuration B (it has been shown that H-bonding between the –NH atom of tryptophan and negatively charged amino acids leads to fluorescence quenching^26^) or by solvent in the case of configuration C. It is notable that the reduction in fluorescence intensity is gradual, consistent with the progressive enrichment of the active site configuration C we observe in the MD simulations of structures between R5 and R7-2. A Trp50Ala mutant of R7-2 was made to compare against R1_Trp50Ala; no change in fluorescence intensity was observed other than ~15% lower intensity at 293 K in R7-2_Trp50Ala compared to R1_Trp50Ala due to the absence of two solvent accessible phenylalanine residues (Phe77Ile, Phe229Ser) (Supplementary Fig. 3, Supplementary Table 1). The Trp50Ala mutants in R1 and R7-2 result in significant (>95%) reductions in *k*_cat_, although the *K*_M_ of R7-2_Trp50Ala increased sevenfold, whereas the *K*_M_ of R1_Trp50Ala was unchanged, suggesting more involvement of Trp50 in substrate binding in the evolved configuration C (Supplementary Table 3).

### One enzyme: three active site configurations

The structural, spectroscopic and computational results suggest that KE07 has evolved through maximizing the sampling of an active site configuration (C). To investigate whether the active site configurations that become enriched by R7-2 are catalytically competent, we performed empirical valence bond (EVB) simulations^27^ of the Kemp elimination, as catalyzed by variants R1 (configuration A), R5 (configurations A and B), R7 (configurations A and B) and R7-2 (configurations B and C). The EVB method describes chemical reactivity within a valence bond framework using classical force fields, and has been successfully applied to the investigation of the Kemp elimination reaction in enzymes^15,28^. The reaction was modeled based on the valence bond states shown in Supplementary Fig. 8 and the resulting calculated activation-free energies, which are in excellent agreement with experiment, are shown in Supplementary Table 4. It is notable that configurations B and C are consistently better than the original, designed configuration (A).

**Fig. 4:**
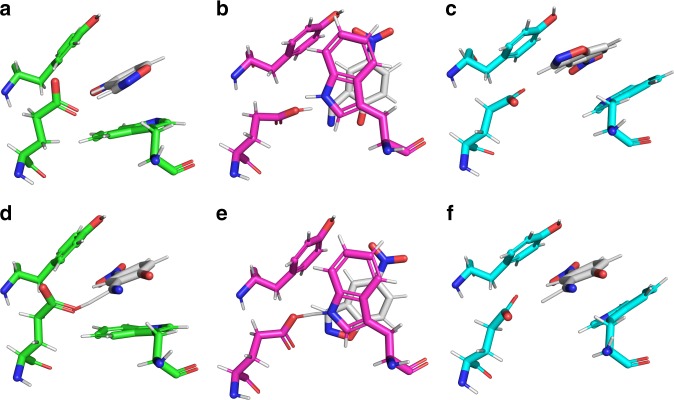
Snapshots of three different binding modes from computer simulations. A comparison of representative structures of the equilibrated **a**−**c** Michaelis complexes and **d**−**f** transition state structures for the Kemp elimination of 5-nitrobenzisoxazole catalyzed by (**a**, **d**, green sticks) R7 with Trp50 in conformation A (**b**, **e**, pink sticks) R7-2 with Trp50 in conformation B, and (**c**, **f**, cyan sticks) R7-2 with Trp50 in conformation C. This figure highlights the shift in substrate position and key interacting residues upon rotation of the Trp50 side chain. The snapshots shown here correspond to the top ranked cluster obtained by RMSD clustering of the EVB simulations, as described in the Methods

Figure 4 shows the substrate positioning relative to key active site residues in the Michaelis complexes and transition states for the reactions catalyzed by the R7 or R7-2 A, B, and C configurations (Supplementary Fig. 9, Supplementary Tables 5-7). Firstly, and most importantly, these calculations confirm that both configurations B and C are similarly catalytically competent, i.e. the configurations are bona fide catalytic states. We observe, as in previous studies^14–17^, that removal of the Glu101-Lys222 salt bridge appears to increase the p*K*_a_/charge of the Glu101 side chain by ~2 p*K*_a_ units (Supplementary Table 5), while the electrostatically unfavorable contribution of Lys222 to the calculated activation-free energy is substantially reduced by the removal of this salt bridge (Supplementary Fig. 10). In the most evolved versions of the three configurations we obtained from our structural studies (R7, A; R7-2, B; R7-2, C), configurations B and C displayed lower energy barriers than A (16.8 and 16.4 vs. 19.0 kcal mol^−1^, respectively; Supplementary Table 4), which is consistent with the gradual conformational selection of state C along the trajectory. The reason for the increased efficiency of state C seems to be an accumulation of many small effects (we will focus on the A:C comparison since C was the state that was primarily selected). First, for all three Trp50 configurations, the substrate position is stabilized through π-stacking interactions with the Trp50 side chain. However, the Trp50:substrate alignment is on average slightly better in conformation C than conformation A (11.3 vs. 14.6°) (Fig. 4; Supplementary Table 7) consistent with the kinetic analysis showing that the Trp50Ala mutant had a greater effect on *K*_M_ in R7 than in R1 (Supplementary Table 3). Second, the orientation of the reactants in state C is also better than state A (donor-H-acceptor angle of 160.4° vs. 149.5°). Finally, the p*K*_a_ of the catalytic Glu101 is predicted to be higher in configuration C than configuration A (7.5 vs. 6.1) owing to differences in the microenvironment of the active site due to the Trp50 rotation, which is again consistent with the experimentally determined changes in p*K*_a_^14^. Altogether, these results support the experimental observations: specifically, catalytically competent active site configurations emerged from disorder and the arrangement of the amino acids in these states (particularly configuration C) was superior to the original design, resulting in evolutionary conformational selection.

### The role of quantum tunneling

Primary kinetic isotope effects (1° KIEs) occur when atoms that are directly involved in the reaction are replaced with heavier isotopes (e.g. hydrogen for deuterium) and can reveal much about the nature of the catalytic mechanism. Using hydrogenated and monodeuterated (at the 3′-hydrogen) 5-nitrobenzisoxazole^29,30^, we determined the activation energy (*E*_a_) and pre-exponential factor (*A*) for the reaction in aqueous solution (Table 1). Given that hydrogen abstraction is the rate-limiting step in the Kemp elimination reaction^11^, it was not surprising that ring opening in the deuterated analog occurred more slowly (Table 1, Supplementary Fig. 11). The relative rates of the hydrogenated and deuterated analogs allow calculation of the 1° KIEs for this reaction, yielding a value of 4.9 at 298 K. To investigate the contribution of quantum tunneling to this 1° KIE, the H abstraction by a hydroxide ion-water cluster (^–^OH•(H_2_O)_4_) was studied from first principles (Supplementary Fig. 12), using quantum chemistry^31^. The free energy activation barrier for H abstraction by the hydroxide ion-water cluster in the water continuum solvent model is consistent with previous work (17.4 vs. 19.8 kcal mol^−1^)^31^. Gas-phase calculations reveal that the H abstraction process is diffusion controlled without explicit H_2_O molecules to stabilize the reactive ^–^OH (Supplementary Table 8, Supplementary Fig. 13). QM/MM calculations were carried out with Polyrate in the H abstraction step of the intermediate complex (with the rate constant *k*_cat_), and the corresponding values of the tunneling coefficients *κ* for the hydrogenated and deuterated molecules between 283 and 323 K are given in Supplementary Table 9. As expected, tunneling is greater for the lighter hydrogen isotope, with a tunneling-corrected KIE of 5.8 for the reaction from the pre-complex at 298 K, and 5.8 at 298 K when starting from the isolated reactants. These results establish that although quantum tunneling is likely to occur in solution and contribute to the magnitude of the KIE, its overall contribution to the reaction rate is likely to be relatively small.

**Fig. 5:**
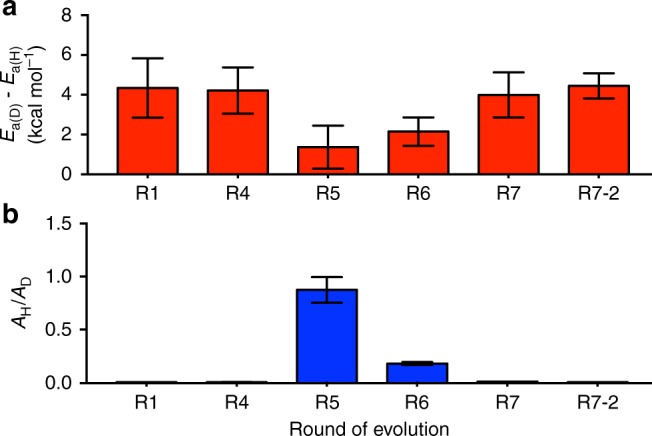
Preorganization and primary kinetic isotope effects in KE07 evolution. Activation energy KIEs (**a**) and pre-exponential factor KIEs (**b**). Error bars denote propagated standard errors in the fitted parameters to Arrhenius plot (Supplementary Fig. 11) from three independent experiments

Given that the theoretical calculations show some level of quantum tunneling in solution, and recent work has implicated quantum tunneling in enzyme catalysis and molecular evolution^8,10^, we investigated the magnitude of the KIE across the trajectory; if enhanced quantum tunneling were selected for throughout evolution, as has been proposed^5^, a substantial increase in the magnitude of the KIEs would be expected. Our results reveal that although there were transient changes to the KIEs in the middle of the evolutionary trajectory (*E*_a(D)_ vs. *E*_a(H)_ and *A*_H_/*A*_D_ values at R5 and R6), the KIEs from the start (R1) and end-point (R7-2) were very similar (Table 1, Fig. 5). Notably, R1 and R7/R7-2 are both relatively conformationally stable, as judged by the molecular dynamics simulations and tryptophan fluorescence spectroscopy. The anomalous KIEs observed in R5−R6 correlate with the increased sampling of the alternative active site configurations (Fig. 5). This is notable because QM/MM calculations have previously shown that, when the conformational coordinate is included in the catalytic model, the presence of distinct active site configurations with different catalytic efficiencies (as observed here) can account for anomalous KIEs without the need to invoke large changes in quantum effects.^4^ It therefore appears that evolution has not involved optimization of short-timescale (fs) vibrations that could enhance quantum tunneling in this example.

## Discussion

In this work, we have followed the iterative improvement of a designed enzyme, dissecting the respective contributions of various effects on catalysis. In addition to traditionally considered effects, such as mutations increasing the basicity of a catalytic residue (e.g., Glu101), our results highlight the significance of catalytic tradeoffs: the same mutational changes that alter active site electrostatics can lead to disorder that is detrimental to catalysis^32^. Other effects, such as quantum tunneling, are not observed to significantly change in this evolutionary process, nor to greatly differ from the level of tunneling that normally occurs in solution.

The most unusual observation in this work was that a series of remote point mutations could completely remodel the designed active site, via subtle changes that involve filling internal cavities and backbone adjustments, leading to a single enzyme that is able to sample three different active configurations, each with different catalytic efficiency. Conformational selection of the most efficient configuration then produced a superior enzyme. Conformational selection has been observed in the optimization of catalytic precision in other designed enzymes^2,19,33^, but the scale of the conformational reorganization observed here is particularly notable. Alongside our KIE data, our results suggest that to accurately model enzyme catalysis by populations of conformationally heterogeneous enzymes, catalytic models should incorporate multiple conformational substates comprising different active site geometries with different catalytic efficiencies^4^.

These results highlight the remarkable conformational plasticity of proteins and the degree to which the configuration of an active site can be modulated by the amino acid composition of the second shell. It is notable that the selection of nondesigned active site conformations through directed evolution appears to be more common when working with computationally designed enzymes^19^. We suggest that this could be due to less focus on the optimization of outer-shell residues to stabilize the active site geometry, in comparison to naturally evolved enzymes in which the composition of the outer-shell has evolved over many generations to stabilize active sites via multiple mechanisms^9^. This study adds to an emerging view in which the conformational plasticity of proteins underpins their remarkable evolutionary potential^34^ and establishes that, provided the active site contains the necessary catalytic machinery, it is the outer-shell amino acids that provide most of the catalytic power of enzymes by positioning the active site correctly for catalysis and allowing it to sample alternative conformations for substrate and product diffusion.

## Methods

### Mutagenesis and protein purification

KE07 variants, cloned into the pET-29b(+) vector (Invitrogen), were expressed with C-terminal His_6_-tags in *Escherichia coli* BL21(DE3) cells (Invitrogen). The cell pellet was resuspended in buffer A (50 mM Tris-HCl pH 8.0, 100 mM NaCl, 20 mM imidazole) and lysed with a French pressure cell press (Thermo Fisher). The soluble fraction was loaded onto a Ni-NTA column (Qiagen) and elution was achieved with buffer B (50 mM Tris-HCl pH 8.0, 100 mM NaCl, 250 mM imidazole). After extensive dialysis against elution buffer (25 mM HEPES-NaOH pH 7.25, 100 mM NaCl), protein concentrations were determined using a NanoDrop spectrophotometer (Thermo Fisher) at 280 nm and SDS-PAGE gel electrophoresis. Extinction coefficients for each of the KE07 variants at 280 nm were calculated using ProtParam^35^. The proteins were concentrated (10–30 mg ml^−^^1^) and stored at 4 °C. Gibson assembly-based mutagenesis^36^ was used to introduce the Trp50Ala substitution into the KE07 R1 and KE07 R7-2 plasmids.

### Kemp elimination kinetics

Kemp elimination of 5-nitrobenzisoxazole (PubChem CID: 142385) results in the formation of 2-cyano-4-nitrophenol (PubChem CID: 11116377). The product has maximum absorbance at 380 nm (molar absorption coefficient of 15,800 M^−1^ cm^−1^ in water)^11^. The rate of product formation was monitored via absorbance at 380 nm using a Cary 60 UV−Vis spectrophotometer (Agilent Technologies) or a SpectraMax-M2 Multi-mode microplate reader (Molecular Devices). Full Michaelis−Menten kinetics were collected at each point. Reaction rates were measured in buffer containing 25 mM HEPES-NaOH pH 7.25, 100 mM NaCl at a range of temperatures (10, 20, 25, 30, 35, 40, 45, 50 °C). To increase substrate solubility 1.25% (v/v) glycerol was included in the assay buffer. Substrate stocks in acetonitrile (final concentration of acetonitrile 1–1.5% v/v) at 9−11 different concentrations (0.01−1.1 mM) were added to the enzyme-buffer mixture immediately before the measurement due to the water sensitivity of the substrates. The rate of the background reaction was subtracted before fitting the data for enzyme-catalyzed reactions to a kinetic model. The activation energies (*E*_a_) and the pre-exponential factors (*A*) were obtained from Arrhenius plots. The plot of ln(*k*) against 1/*T* gives a straight line with slope –*E*_a_/*R* and *y*-intercept ln(*A*), according to the linear form of the Arrhenius equation (Eq. 1).

1 $${\mathrm{ln}}\,\left( k \right) = {\mathrm{ln}}\left( A \right) - \frac{{E_{\mathrm a}}}{R}\left( {\frac{1}{T}} \right),$$

where *A*, *R*, *k*, and *T* are pre-exponential factor, gas constant, reaction rate, and temperature in Kelvin, respectively. The activation energy *E*_a_ was calculated from the slope of the Arrhenius plot. *A* is calculated from the *y*-intercept of the plot.

### Substrate synthesis

5-nitrobenzisoxazole was prepared from 1,2-benzisoxazole (Sigma, >99%) following the published protocol^37^: 2 ml (2.3 g) of 1,2-benzisoxazole was dissolved in concentrated H_2_SO_4_ (20 ml) in a flask (placed on the salt−ice mixture), then 2 ml mixture of H_2_SO_4_ (1 ml) and HNO_3_ (3 ml) was slowly added to the 1,2-benzisoxazole mixture. The solution was stirred for 30 min. Fifty milliliters of ice−water mixture was added to the stirred mixture and the filtered crude product was recrystallized by solvating it to heated anhydrous ethanol (70–80 °C) then slowly cooled and filtered. The yield of 2 days vacuum-dried product was 3.3 g. 3-Deutero-5-nitrobenzisoxazole was synthesized from 2-bromophenol following the published procedures^29,30^. The substrate synthesis scheme is shown in Supplementary Fig. 14 and consists of four steps:

(1) - 2-(2-Bromophenoxy)tetrahydro-2H-pyran: Pyridinium p-toluene sulfonate (754 mg, 3.00 mmol) was added to a solution of 2-bromophenol (3.48 ml, 30.00 mmol) and 2,3-dihydropyran (4.11 ml, 45.00 mmol) in dry CH_2_Cl_2_ (20 ml). The resulting mixture was stirred for 21 h under an atmosphere of nitrogen at room temperature. Thereafter, the reaction mixture was quenched with sat. aq. NaHCO_3_ (20 ml) and the resulting phases separated. The aqueous phase was then back-extracted with CH_2_Cl_2_ (3 × 20 ml) and the organic phases were combined, dried over Na_2_SO_4_, filtered and the filtrate concentrated in vacuo. The residue was adsorbed to silica and purified by flash chromatography (SiO_2_, EtOAc:Hex (1:10)) to give 1 (6.61 g, 86%) as a colorless oil. ^1^H NMR (400 MHz, CDCl_3_) *δ*_H_ 7.54 (dd, *J* = 7.8, 1.6 Hz, 1 H), 7.24 (ddd, *J* = 7.4, 7.4, 1.6 Hz, 1 H), 7.16 (dd, *J* = 8.6, 1.2 Hz, 1 H), 6.87 (ddd, *J* = 7.8, 7.4, 1.6 Hz, 1 H), 5.53 (t, *J* = 2.7 Hz, 1 H), 3.92 (ddd, *J* = 11.0, 3.1 Hz, 1 H), 3.67−3.57 (m, 1 H), 2.19−2.05 (m, 1 H), 2.04−1.95 (m, 1 H), 1.94−1.83 (m, 1 H), 1.80−1.59 (m, 3 H). ^13^C NMR (100 MHz, CDCl_3_) *δ*_C_ 153.4, 133.2, 128.3, 122.7, 116.6, 113.1, 96.6, 61.8, 30.2, 25.2, 18.3. MS (EI^+^) m/z 258 (10%, [M, ^81^Br]^+^), 256 (10%, [M, ^79^Br]^+^).

(2) - 2-α-Deuterio-(tetrahydropyran-2-yloxy)benzaldehyde: A solution of 2.2 M n-BuLi in hexanes (4.62 ml, 10.27 mmol) was added dropwise to a solution of 1 (2.64 g, 10.27 mmol) in dry Et_2_O (20 ml) at 0 °C under an atmosphere of nitrogen. The resulting mixture was allowed to stir for 2 h at 0 °C. Thereafter, a solution of [D^7^]-DMF (879 μl, 11.29 mmol) in dry Et_2_O (5 ml) was added and the solution was stirred, allowing to warm to room temperature for 17 h. Thereafter, the reaction was quenched by addition of D_2_O (20 ml) and the resulting layers were separated. The aqueous phase was back-extracted with Et_2_O (3 × 15 ml) and the organic layers were combined, dried over Na_2_SO_4_, filtered and the filtrate concentrated in vacuo. The residue was purified by flash chromatography (SiO_2_, Et_2_O:Hex (1:3)) to give 2 (2.01 g, 94%) as a colorless oil. ^1^H NMR (400 MHz, CDCl_3_) *δ*_H_ 7.85 (s, 1 H), 7.56−7.50 (m, 1 H), 7.27−7.23 (m, 1 H), 7.10−7.04 (m, 1 H), 5.59 (t, *J* = 2.7 Hz, 1 H), 3.94−3.85 (m, 1 H), 3.72−3.62 (m, 1 H), 2.09−1.87 (m, 3 H), 1.83−1.56 (m, 3 H). ^13^C NMR (100 MHz, CDCl_3_) *δ*_C_ 175.8, 159.5, 135.8, 128.0, 121.5, 115.4, 96.4, 62.1, 30.1, 25.0, 18.5. MS (EI^+^) m/z 207 (5%, [M]^+^), 123 (100%, [M-THP]^+^).

(3) - α-Deuterio-2-hydroxybenzaldehyde: A solution of 1 M DCl in D_2_O (10 ml) was added to a solution of 2 (2.01 g, 9.91 mmol) in dry THF (10 ml) under an atmosphere of nitrogen and the resulting solution was stirred for 17 h. Thereafter, the reaction mixture was diluted with D_2_O (10 ml) and the resulting mixture extracted with Et_2_O (3 × 20 ml). The organic layers were combined, dried over Na_2_SO_4_, filtered and the filtrate concentrated in vacuo. The residue was purified by flash chromatography (SiO_2_, EtOAc:Hex (1:10)) to give **3** (915 mg, 75%) as a colorless oil. ^1^H NMR (400 MHz, CDCl_3_) *δ*_H_ 11.05 (s, 1 H), 7.06–7.51 (m, 2 H), 7.06-6.97 (m, 2 H). ^13^C NMR (100 MHz, CDCl_3_) *δ*_C_ 196.5, 161.7, 137.0, 133.7, 119.8, 117.6. MS (EI^+^) m/z 123 (100%, [M]^+^).

(4) - 3-Deuterio-5-nitrobenzo[d]isoxazole: Hydroxylammonium O-sulfonate (966 mg, 8.54 mmol) was added to a solution of **3** (700.8 mg, 5.69 mmol) in EtOH (3.5 ml) under an atmosphere of nitrogen and the resulting solution was stirred for 30 min (over which time the mixture clarified). CH_2_Cl_2_ (15 ml) was added and the mixture was cooled to 0 °C and a solution of NaHCO_3_ (1.05 g, 12.49 mmol) in D_2_O (7 ml) was added dropwise with the evolution of gas and the resulting mixture was stirred for a further 30 min. Thereafter, the resulting layers were separated and the aqueous layer back-extracted with CH_2_Cl_2_ (4 × 10 ml). The organic layers were combined, dried over Na_2_SO_4_, filtered and the filtrate concentrated in vacuo to give a colorless oil that was used without further purification. A mixture of HNO_3_ (0.4 ml) and H_2_SO_4_ (0.15 ml) was added to a solution of the colorless oil (701 mg, 5.83 mmol) in H_2_SO_4_ (3.6 ml) at 0 °C under an atmosphere of nitrogen. The resulting solution was stirred for 30 min and then was carefully poured into a mixture of ice and water (50 ml, 1:1). The resulting precipitate was collected and recrystallized from dry EtOH to give the final product (273 mg, 28% over two steps) as a colorless solid. ^1^H NMR (400 MHz, CDCl_3_) *δ*_H_ 8.73 (d, J = 2.0 Hz, 1 H), 8.52 (dd, *J* = 9.2, 2.0 Hz, 1 H), 7.77 (d, *J* = 9.2 Hz, 1 H). ^13^C NMR (100 MHz, CDCl_3_) *δ*_C_ MS (EI^+^).

### X-ray crystallography

Crystals of all KE07 variants except one (R7-2 with product) were grown at 4 °C by hanging-drop vapor diffusion. Equal volumes of reservoir solution (12–25% PEG 3350, 0.1 mM Bis-Tris Propane pH 8.5) or (25 mM HEPES-NaOH pH 7.25, 100 mM NaCl) were mixed with protein (5–30 mg ml^−1^) and crystals reached maximum size after 7–60 days of incubation. One of the R7-2 crystals was grown by mixing 2 μl of reservoir solution (0.5 M (NH_4_)_2_SO_4_, 0.8 M Li_2_SO_4_, 0.1 M Na_3_citrate pH 5.6) and 1 μl of protein (11 mg ml^−1^), the crystals reached maximum size after 1–2 months at 18 °C. 35% polyethylene glycol (PEG400), 35% 2-methyl-2,4-pentanediol (MPD), 35% glycerol or 35% PEG3350 were used as a cryoprotectant. Crystals were soaked in a solution containing 35% MPD and 0–7.5 mM 5-nitrobenzisoxazole and for between 10 s and 40 min before vitrification in nitrogen at 100 K. Crystallographic data were collected at 100 K at the Australian Synchrotron (MX1/MX2, 0.9537 Å) except (5D2V) (Australian National University (MarμX, 1.5418 Å)). The obtained diffraction data were indexed and integrated with XDS^38^. Resolution estimation and data truncation were performed on the basis of the datasets overall half-dataset correlation, a CC_1/2_ value of 0.3–0.5^39^. All structures were solved by molecular replacement using the Molrep program in CCP4^40^ using either the structure deposited under PDB accession code(2RKX) or (3IIV) as a starting model. The models were refined using phenix.refine^41^, and the model was subsequently optimized by iterative model building with the program COOT v0.7^42^. The alternative conformations were modeled based on m*F*_o_-D*F*_c_ density and the occupancies and B-factors were determined using phenix.refine^41^. The structures were then evaluated using MolProbity in Phenix. Details of the refinement statistics were produced by Phenix v1.9^43^ and summarized in Supplementary Data 1. Figures were made using PyMol v1.7^44^.

### Fluorescence measurements

Tryptophan fluorescence was measured using a Cary Eclipse Fluorescence Spectrophotometer with a Single Cell Peltier Accessory for temperature control (Agilent Technology). Tryptophan was excited at 280 nm (slit width 5 nm) and emission was monitored between 300 and 400 nm (1.5 nm slit width). Purified KE07 protein was diluted (1.8–5 μM) in buffer (25 mM HEPES pH 7.25, 100 mM NaCl) and the spectra were measured at 283–323 K.

### Computer simulations

HREX-MD simulations: HREX-MD simulations were performed in GROMACS 5.1.4^45^ patched with PLUMED 2.3.0^46^. The simulations were initiated from the following starting structures: PDB IDs (5D2W) for R1,(3IIO)^14^ chain A for R4,(5D30) for R5,(5D32) chain A for R6, (5D33) chain A for R7, and (5D38) chain A for R7-2. The His-tags were removed, and the protonation states for titratable residues were set according to PROPKA^22^ calculations at pH of 7.25, i.e., all Asp and Glu were deprotonated, Lys and Arg protonated, and His residues were neutral. The protein, modeled with the AMBER 99SB*-ILDN force field^47,48^, was centered in a dodecahedral box with the edges at least 10 Å away from the protein. The system was solvated with TIP3P water molecules^49^ and the net charge was neutralized with sodium ions. Periodic boundary conditions were imposed, where the long-range electrostatic interactions were calculated using the particle mesh Ewald method^50^. The short-range nonbonded interactions were calculated under the cutoff of 10 Å. All bonds were constrained using the LINCS algorithm^51^, and a 2 fs integration time step was used. The minimized system (2000 steps, steepest descent algorithm) was gradually heated and equilibrated for 0.2 ns in the NVT ensemble (at 298 K, velocity-rescaling thermostat)^52^, where the protein atoms were restrained using a force constant of 1000 kJ mol^−1^ nm^−2^. In the following 2.5 ns long NPT equilibration (at 1 bar, Berendsen barostat^53^), the restraints were gradually reduced to 5 kJ mol^−1^ nm^−2^. The unrestrained production HREX-MD was performed under constant temperature and pressure, using the velocity-rescaling thermostat and the Parrinello–Rahman barostat^54^.

Six replicas were used in each HREX-MD simulation, where the hot region^55^ included Trp50 and residues in a 4 Å sphere around it (i.e., 9–11, 48–52, 80–81, 101, 128, 201, and 222). The Hamiltonian scaling factors for the nonbonded interactions and proper dihedrals in the hot region were 1.000, 0.922, 0.850, 0.784, 0.723, and 0.667. The exchange between replicas was attempted every 4 ps during 200 ns simulations, giving the average exchange acceptance ratio of 35–40%. The *χ*-angles used for the analysis were extracted every 2.5 ps from the unperturbed replica (i.e., the one with the Hamiltonian scaling factor of 1.0). The backbone RMSD profiles for all unperturbed replicas are shown in Supplementary Fig. 7. The accumulated sampling time for one HREX-MD simulation was 1.2 μs, and 7.2 μs over all systems.

Empirical valence bond simulations: Empirical valence bond simulations were performed using Q^56^, version 5.10, and the OPLS-AA force field^57^. All non-standard force field parameters used to describe the substrate and product molecules were obtained using Macromodel 9.1, version 11^58^. The following crystal structures were simulated: (4Z08), and (5D2W) for R1 (both with Trp50 in conformation A), (5D30) with Trp50 in low occupancy (conformation A) and high occupancy (conformation B) for R5,(6DC1) chain A and (5D33) chain A for R7 (Trp50 conformations A and B, respectively), and (5D38) chain A and (6CT3) chain A for R7-2 (Trp50 conformations B and C, respectively). The 5-nitrobenzisoxazole substrate was either overlaid with the product crystallized in the active site, or in the absence of a product manually placed in the active site, in such a way as to optimize the alignment of the donor-hydrogen-acceptor between the substrate and the Glu101 side chain, as well as to maximize stabilizing interactions from the surrounding residues. The entire system was then solvated in a 20 Å radius of TIP3P water molecules^49^, subject to surface-constrained all-atom solvent (SCAAS) boundary conditions^56,59^, and centered on the C_δ_ atom of Glu101. The system was modeled using a multilayer approach, in which all atoms within the inner 85% of this water droplet were allowed to move freely, the atoms in the external 15% of the droplet were restrained to their crystallographic positions using a 10 kcal mol^−1^ Å^−2^ harmonic restraint, and all atoms outside the droplet were fixed at their crystallographic positions using a 200 kcal mol^−1^ Å^−2^ restraint. All residues within the mobile region (i.e., the inner 85%) were protonated based on examination of expected protonation states using PROPKA 3.1^22^, and all residues falling outside the mobile region were kept in their neutral forms to avoid introducing system instabilities due to the presence of charged residues outside the water droplet (note that this does not introduce structural instabilities as these atoms are restrained to their crystallographic positions).

All simulations were performed using the Berendsen thermostat^53^ with the leapfrog integrator, and with the solute and solvent coupled to individual heat baths. A 10 Å cutoff was used for the calculation of nonbonded interactions (with the exception of reacting atoms, which were subject to a 99 Å cutoff, *i.e.,* essentially no cutoff), and electrostatic interactions for all atoms falling beyond this cutoff were approximated using the local reaction field approach^60^, with a nonbonded pairlist update every 30 fs. The bonds to hydrogen atoms were constrained using the SHAKE algorithm^61^. All systems were subjected to an initial 3 ps minimization at 1 K and 0.1 fs step size to remove bad contacts in the system, during which time a 200 kcal mol^−1^ Å^−2^ harmonic restraint was applied to all solute atoms to keep them to their crystallographic positions. The step size was then increased to 1 fs for the remainder of the simulation time, and the temperature was gradually increased to 300 K while dropping the restraint to 0.5 kcal mol^−1^ Å^−2^ on only the reacting atoms (not taking into account the restraint on atoms outside the mobile region), over a total of 210 ps of simulation time. Once each system had been heated to 300 K, it was subjected to a further 30 ns of equilibration. Each equilibration was performed three times with three different sets of initial velocities, leading to 90 ns of equilibration time per system, and 720 ns of equilibration time over all systems. The corresponding backbone root mean square deviations are shown in Supplementary Fig. 15.

For each system, the endpoints of the three equilibration runs were then used as starting structures for the subsequent EVB simulations^27^. Three additional equilibration runs of 500 ps in length were performed from each of these starting points, using random velocities, in order to generate nine discrete starting points for our EVB simulations of each system. Our EVB calculations were performed using a simple two-state model, using the valence bond states described in Supplementary Figs. 8 and 16 (see also refs. ^15,28^). The EVB free energy perturbation/umbrella sampling (EVB-FEP/US) calculations were performed in 51 individual mapping frames of 100 ps simulation length per frame, leading to a total of 5.1 ns simulation time per individual EVB trajectory, 45.9 ns simulation time per system, and 367 ns simulation time over all systems (in addition to the equilibration time leading to a total simulation time of 1.09 μs).

The EVB parameters were calibrated using the uncatalyzed background reaction in aqueous solution as a baseline, which was modeled using the 4-nitrobenzisoxazole substrate and propionate as a model for Glu101 (again, see also ref. ^28^). All simulations of this reference state were performed using the same protocol as for the corresponding enzymatic reaction, with the exception that a larger harmonic restraint of 1.0 kcal mol^−1^ Å^−2^ was placed on the reacting atoms to stop them from drifting out of the simulation sphere. The EVB off-diagonal element and gas-phase shift, which are described in detail in e.g. ref. ^27^, were adjusted to reproduce an activation-free energy of 21.2 kcal mol^−1^ in aqueous solution based on the calibration provided in refs. ^15,28^, and the same EVB parameters were then used unchanged to model the reaction in all enzyme variants. This then provides a common reference point to compare the relative energies of all enzyme variants to each other. All EVB parameters used in this work are provided in Supplementary Tables 10 to 21 (Supplementary Fig. 16). Finally, all energy analyses were performed using the Q simulation package and Qtools 0.5.10 ^62^, the RMSD and clustering analyses were performed using GROMACS^45^ (the clustering algorithm described by Daura et al.^63^, with the cutoff of 0.5 Å for the protein atoms), and the geometry analysis was performed using the MDTraj library^64^.

### Computational tunneling evaluation in the Kemp elimination

All molecular orbital theory and density functional theory calculations were carried out with Gaussian 09 software package^65^. Geometries and frequencies of all species were calculated using M06-2X functional. All species were optimized in vacuo or in a field of solvent using SMD/M06-2×/6–31 + G(d,p) method^66^ with water. All transition state structures were characterized with a single imaginary frequency and minimum with zero imaginary frequencies. The tunneling probability was evaluated using the reaction-path variational transition state theory with multidimensional tunneling (RP-VTST/MT) method^67^. These calculations were performed with Polyrate 2010-A via the Gaussrate 2009-A interface to Gaussian 09 ^65^. Gaussian archive entries of the optimized geometries are provided in Supplementary Note 1.

## Electronic supplementary material

Supplementary Information

Description of Additional Supplementary Files

Supplementary Data 1

## Data Availability

The crystal structures of KE07 variants, with and without ligand, have been deposited in the Protein Data Bank under accession codes 6C7H, 4Z08, 5D2T, 5D2V, 5D2W, 6C7V, 6C7M, 6DNJ, 6C7T, 6C8B, 5D30, 5D32, 5D33, 6CAI, 6D31, 5D38, 6CT3. PDB validation reports are all available at www.rcsb.org. All relevant data are available from the corresponding author upon request.
