# Supplementary material for: The evolution of multiple active site configurations in a designed enzyme
Source: Nat Commun. 2018 Sep 25;9:3900. doi: 10.1038/s41467-018-06305-y (PMC6156567; doi:10.1038/s41467-018-06305-y)
Supplement: Supplementary file 1 — Supplementary Information [file 41467_2018_6305_MOESM1_ESM.pdf]

Supplementary Information

The evolution of multiple active site configurations in a designed enzyme

Nan-Sook Hong[1], Dušan Petrović[2], Richmond Lee[1], Miha Purg[2], Ganna Gryn'ova[1,3], Jake Saunders[1], Paul Bauer[2], Paul D. Carr[1], Ching-Yeh Lin[1], Peter D. Mabbitt[1], William Zhang[1], Timothy Altamore[1], Chris Easton[1], Michelle L. Coote[1], Shina C.L. Kamerlin[2] and Colin J. Jackson[1]*

[1] Research School of Chemistry, Australian National University, Canberra, ACT, 2601, Australia

[2] Department of Chemistry, BMC, Uppsala University, Box 576, 751 23 Uppsala, Sweden

[3] Institut des Sciences et Ingénierie Chimiques, École Polytechnique Fédérale de Lausanne, CH-1015 Lausanne, Switzerland

* Correspondence may be addressed to Colin Jackson (colin.jackson@anu.edu.au) or Lynn Kamerlin (lynn.kamerlin@kemi.uu.se).

Supplementary Figures

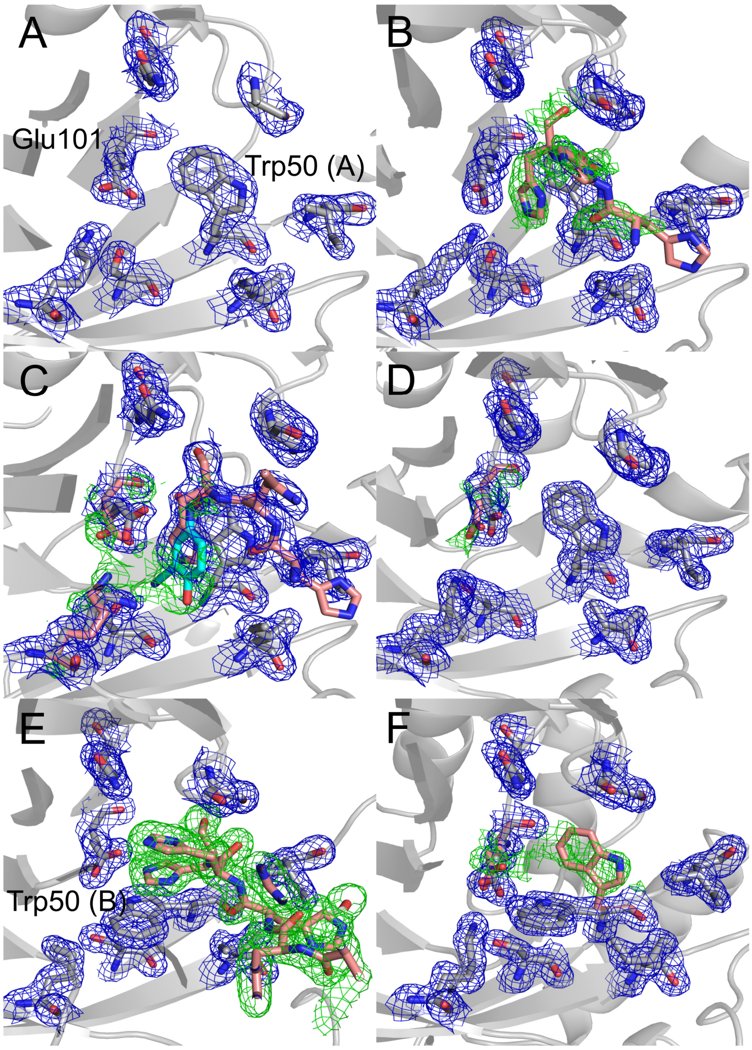

Supplementary Figure 1. Crystal structures of KE07 variants without and with ligand. (**A**) R1 in configuration A with no soaking (pH 7.25). (**B**) R1 in configuration A with bound hexahistidine tag from neighboring protein chain at pH 8.5. (**C**) R1 in configuration A with a mixture of product and hexahistidine tag at pH 8.5 after substrate soaking. (**D**) R5 in configuration A after cryoprotection in glycerol. (**E**) R6 in configuration B with bound hexahistidine tag from neighboring protein chain. (**F**) R7 with mixed occupancy (A/B) after cryoprotection. Ligands and alternative conformations of residues are shown as pink sticks, except product (cyan stick). The mF_o-DF_c omit maps are shown as green meshes and contoured at 2.0σ. The 2mF_o-DF_c maps are shown as blue meshes and contoured at 1.0σ. PDB ID of the structures: **A** (5D2V), **B** (4ZO8), **C** (5D2T), **D** (6C7V), **E** (6C8B), **F** (5D33).

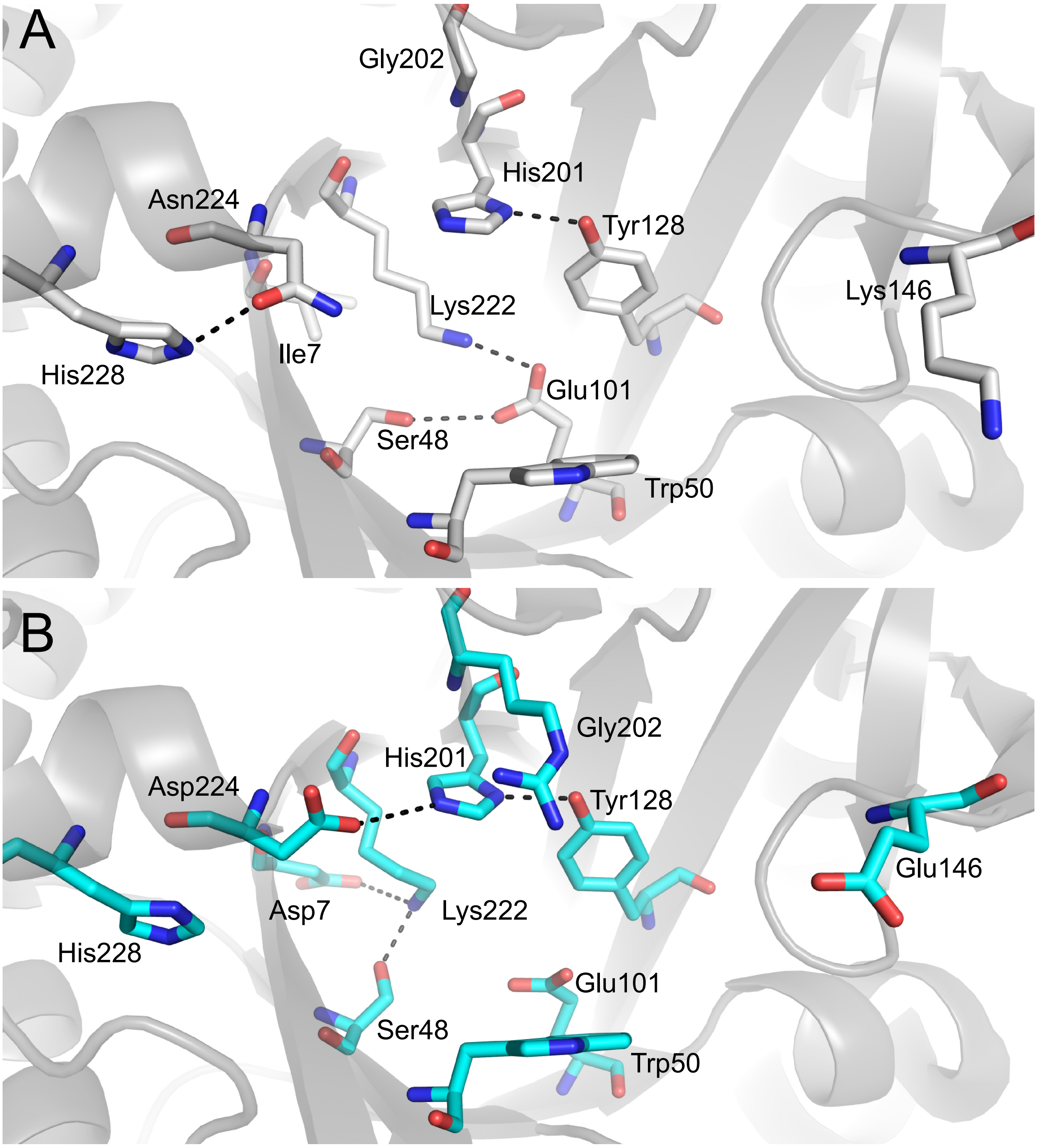

Supplementary Figure 2. Comparison between the active sites of KE07 R1 and R4. Crystal structures of KE07 R1 (**A**) and R4 (**B**) are shown as cartoon and stick representations. The Ile7Asp mutation results in loss of the Lys222-Glu101 salt bridge, as well as the hydrogen bond between Ser48 and Glu101. The Asn224Asp mutation results in a change in the conformation of this residue, favoring His201, rather than His228, as a H-bond partner. The Gly202Arg mutation results in Arg202 extending into the active site, stacking above His201, which is involved in a H-bond network with Asp224 and Tyr128. The Lys146Glu mutation has no obvious effect on the active site. Interactions between side chains are shown as dotted lines.

A

	R1	R4	R5	R6	R7	R7-2
Trp	50<br>156	50<br>156	50<br>156	50<br>156	50<br>156	50<br>156
Tyr	128<br>143<br>240<br>(39)<br>(182)	128<br>143<br>240<br>(39)<br>(182)	128<br>143<br>240<br>(39)<br>(182)	128<br>143<br>240<br>(39)<br>(182)	**+84**<br>128<br>143<br>240<br>(39)<br>(182)	128<br>143<br>240<br>(39)<br>(182)
Phe	23<br>38<br>141<br>229<br>189<br>138<br>77<br>120<br>86<br>(49)<br>(210)<br>(214)<br>(~~227~~)	23<br>38<br>141<br>229<br>189<br>138<br>77<br>120<br>86<br>(49)<br>(210)<br>(214)<br>(227)	23<br>38<br>141<br>229<br>189<br>138<br>77<br>120<br>86<br>(49)<br>(210)<br>(214)<br>(227)	23<br>38<br>141<br>229<br>189<br>138<br>77<br>120<br>86<br>(49)<br>(210)<br>(214)<br>(227)	23<br>38<br>141<br>~~229~~<br>189<br>138<br>~~77~~<br>120<br>86<br>(49)<br>(210)<br>(214)<br>(227)	**+102**<br>23<br>38<br>141<br>~~229~~<br>189<br>138<br>~~77~~<br>120<br>86<br>(49)<br>(210)<br>(214)<br>(227)

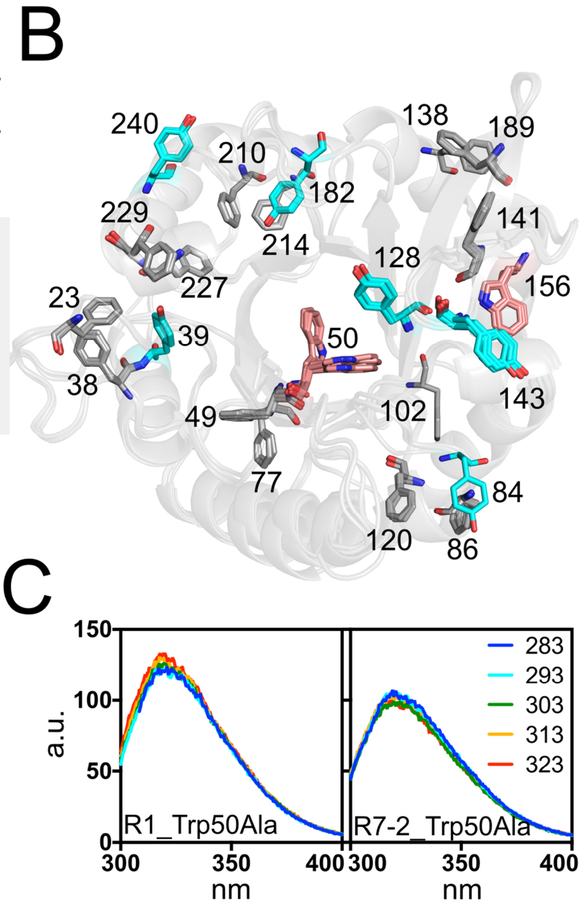

Supplementary Figure 3. Aromatic residues of KE07 variants and tryptophan fluorescence spectra of Trp50Ala mutants of R1 and R7-2. (**A**) Residue numbers of aromatic residues from KE07 variants are listed. Mutations that introduce new aromatic residues are shown in bold in red. Residues that were mutated to non-aromatic residues are indicated in red with strikethrough. Non-solvent accessible residues are in brackets. (**B**) Superimposed aromatic residues, including tryptophan (pink), tyrosine (cyan), and phenylalanine (grey), are shown as sticks on the grey cartoons. (**C**) Fluorescence emission spectra (excitation at 280 nm) of KE07 Trp50Ala mutants of R1 and R7-2 (1.8-5.0 µM) were measured at 283-323 K (temperatures for each spectrum are indicated with different colors).

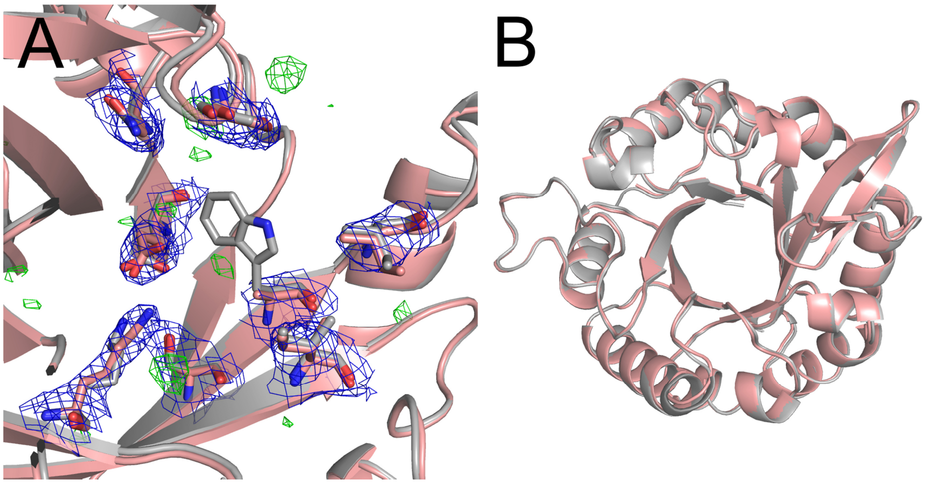

Supplementary Figure 4. Comparison between the KE07 R1 and R1 Trp50Ala mutant. R1 (grey) and R1_Trp50Ala mutant (pink) are superimposed and shown as (**A**) stick and cartoon representations and (**B**) cartoon representations. The 2mF_o-DF_c maps of R1_Trp50Ala are shown as blue meshes and contoured at 1.0σ. The mF_o-DF_c maps are shown as green meshes within 10 Å from the centre (Trp50) and contoured at 3.0σ. PDB ID of the structures: Grey (5D2V), Pink (6C7H).

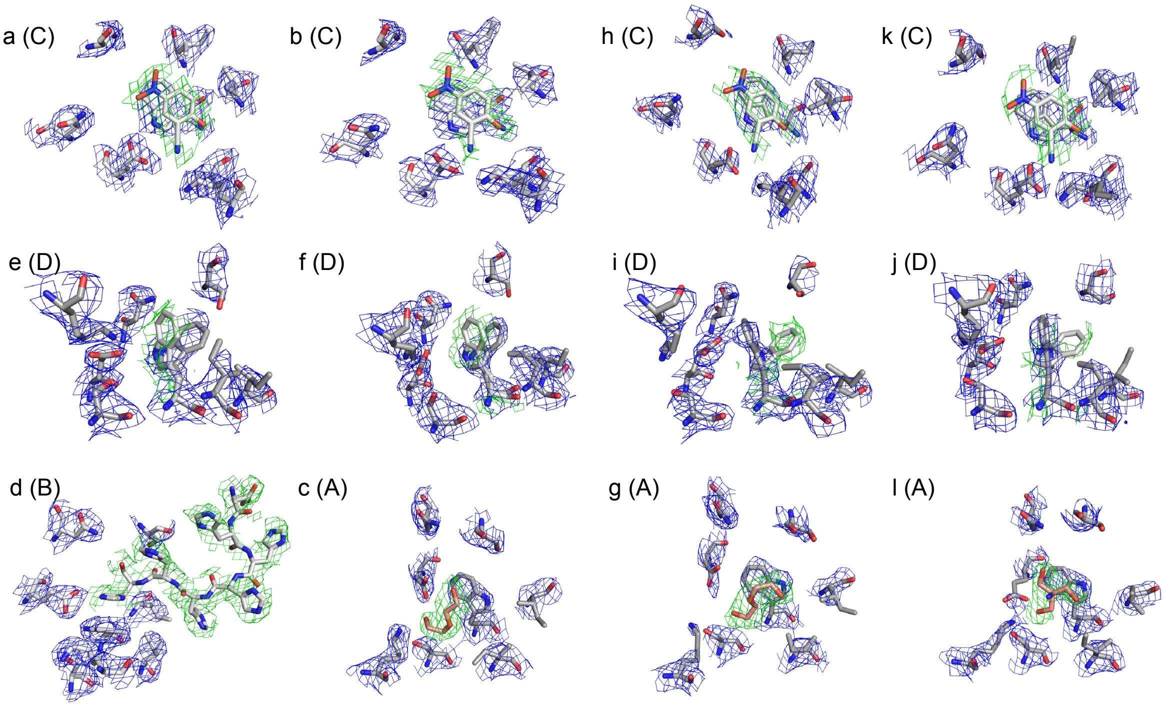

Supplementary Figure 5. Crystal structure of KE07 R7 with ligand. The active site of 12 chains of KE07 R7 are presented with cartoon and stick representations. Chain IDs (a-l) are presented with Trp50 conformation in brackets (A, B, C, D) for each structure. The mF_o-DF_c omit maps are shown as green meshes for ligands and alternative conformations and contoured at 3.0σ. The 2mF_o-DF_c maps are shown as blue meshes and contoured at 1.0σ. PDB ID of the structure: 6DCI.

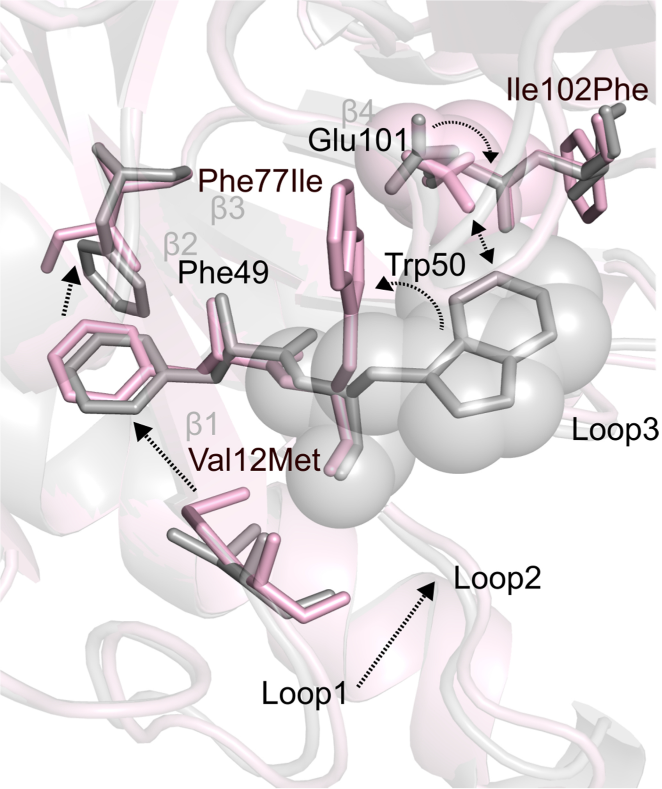

Supplementary Figure 6. Conformational sub-states regulated by second shell mutations in the later rounds of KE07 evolution. R1 (grey) and R7-2 (pink) are superimposed. The second shell mutations, Val12Met, Phe77Ile, result in slight changes to internal hydrophobic cavities, resulting in movement of Phe49 and a change in the backbone torsion angle to favor the new Trp50 orientation. The Ile102Phe mutation results in phenylalanine filling a hydrophobic cavity, adjusting the neighboring backbone conformation and allowing rotation of Glu101, which in turn removes steric hindrance to Trp50 adopting the "flipped" conformation. PDB ID: 5D2W (R1, grey), 5D38 (R7-2, pink).

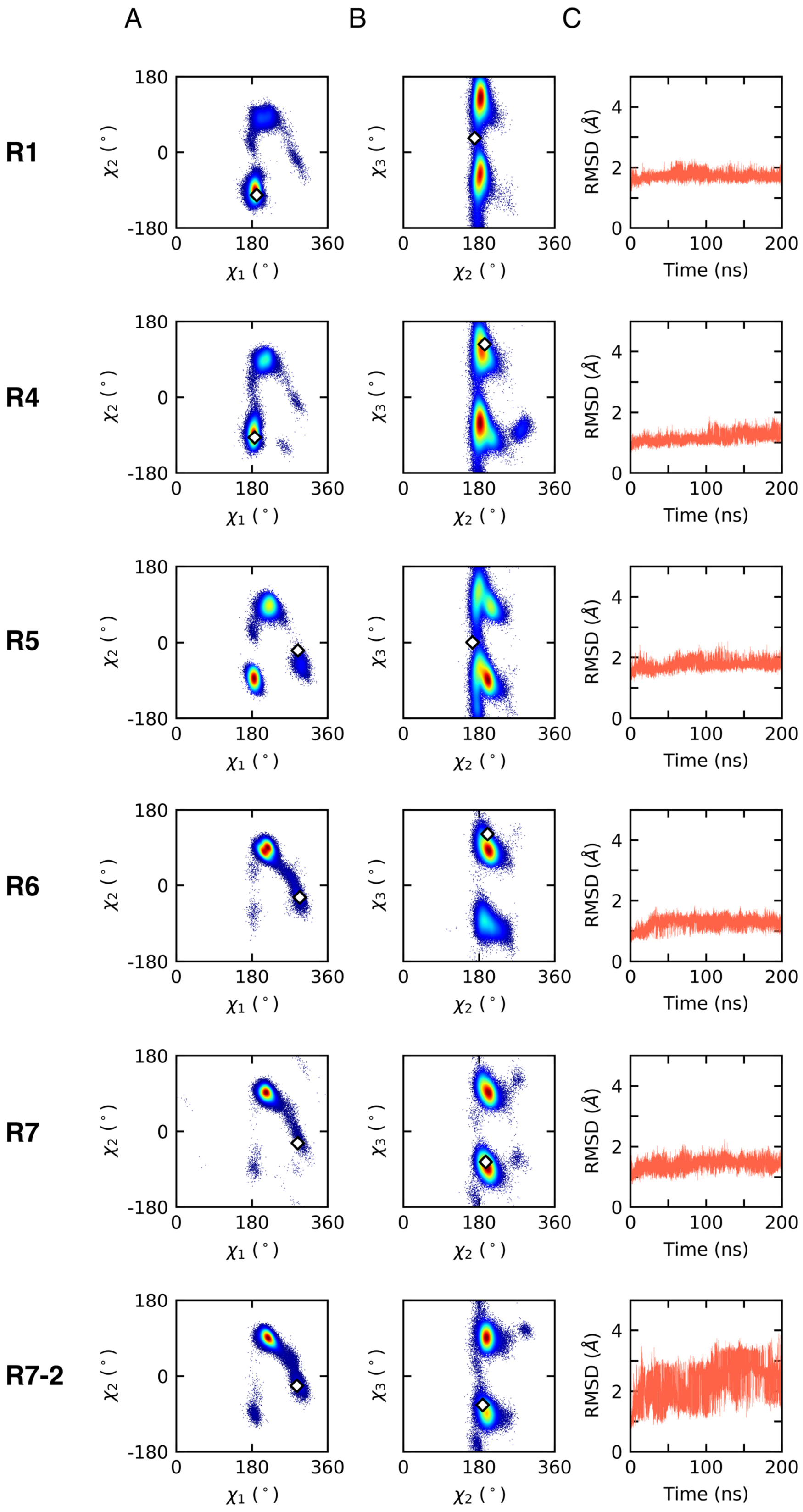

Supplementary Figure 7. Conformational sampling using HREX-MD simulations. Conformational sampling of the (**A**) Trp50 and (**B**) Glu101 side chains during the evolution of KE07, together with (**C**) the corresponding root mean square deviations (RMSD) of backbone atoms during the simulations. The starting values of dihedral angles are designated on the plot by the white diamonds.

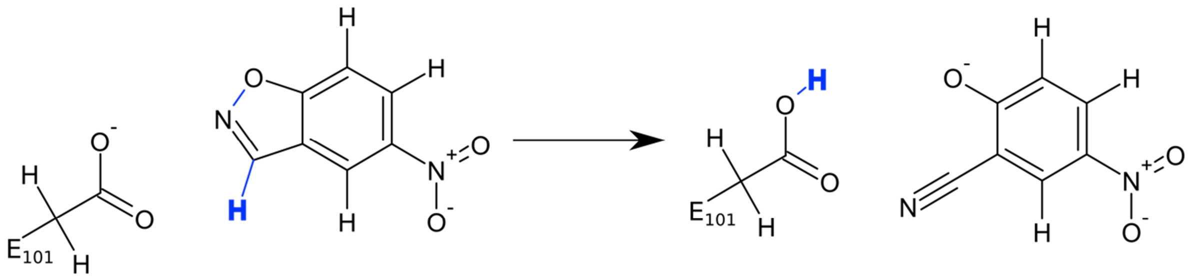

Supplementary Figure 8: Overview of the valence bond states used in our empirical valence bond simulations. This was used to describe the Kemp elimination of 5-nitrobenzisoxazole by the R1 (configuration A), R5 (configurations A and B), R7 (configurations A and B) and R7-2 (configurations B and C) variants.

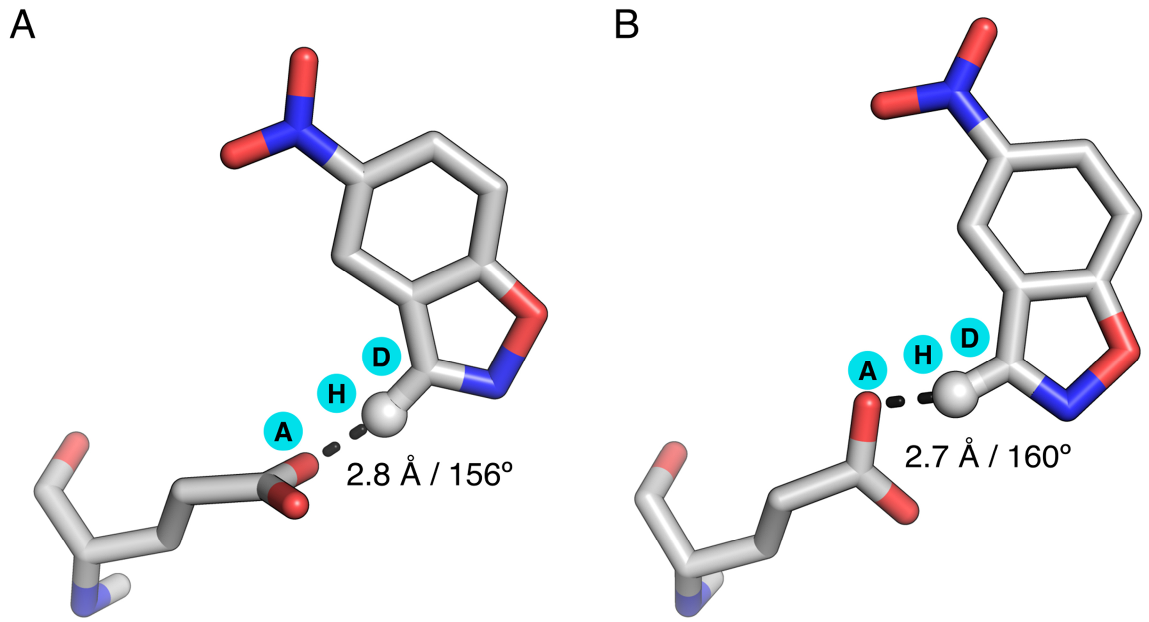

Supplementary Figure 9: Changes in the average D-A distance and D-H…A angle upon moving from R1 to R7-2. The structures shown here are the top ranked clusters of the Michaelis complexes of (**A**) R1 and (**B**) R7-2 obtained from our empirical valence bond simulations by RMSD clustering, and the annotated distances and angles are average values based on the data presented in Supplementary Tables 5-6.

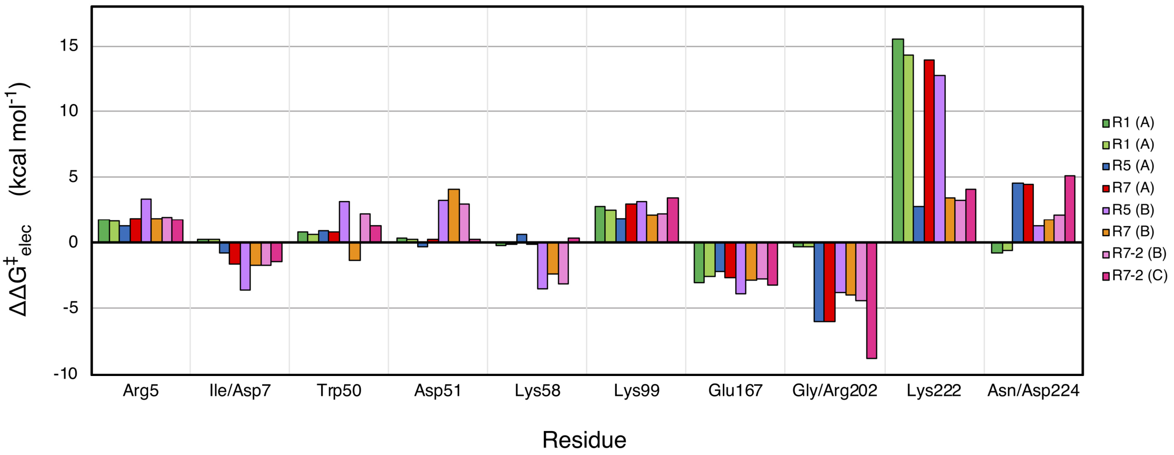

Supplementary Figure 10. Average electrostatic contributions of individual amino acids to the calculated activation free energies ($\Delta\Delta G^{\ddagger}_{elec}$) of the Kemp elimination of 5-nitrobenzisoxazole by the KE07 variants. The activation free energies were calculated using the empirical valence bond (EVB) approach, as described in the Methods, and the different variants are ordered by the Trp50 conformation. All data is shown as averages over 9 independent EVB simulations for the corresponding crystal structure and the Trp50 conformation. The two R1 simulations were initiated from crystal structures 4Z08 and 5D2W, respectively. All energies are shown in kcal mol^{-1}.

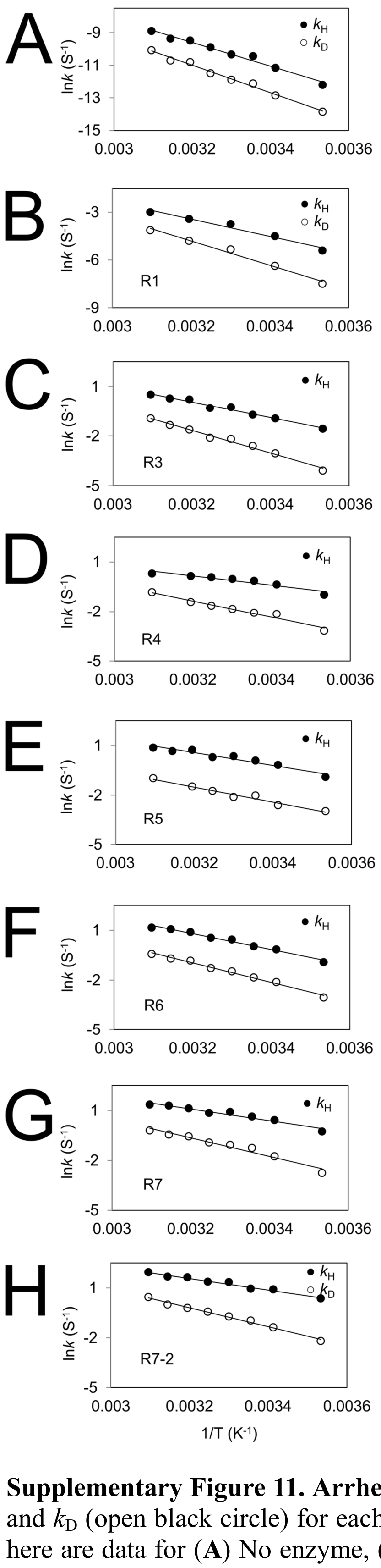

Supplementary Figure 11. Arrhenius plots and KIEs. The Arrhenius plots of k_H (filled black circle) and k_D (open black circle) for each KE07 variant, fitted to the Arrhenius equation (solid line). Shown here are data for (**A**) No enzyme, (**B**) KE07 design R1, (**C**) R3, (**D**) R4, (**E**) R5, (**F**) R6, (**G**) R7, and (**H**) R7-2.

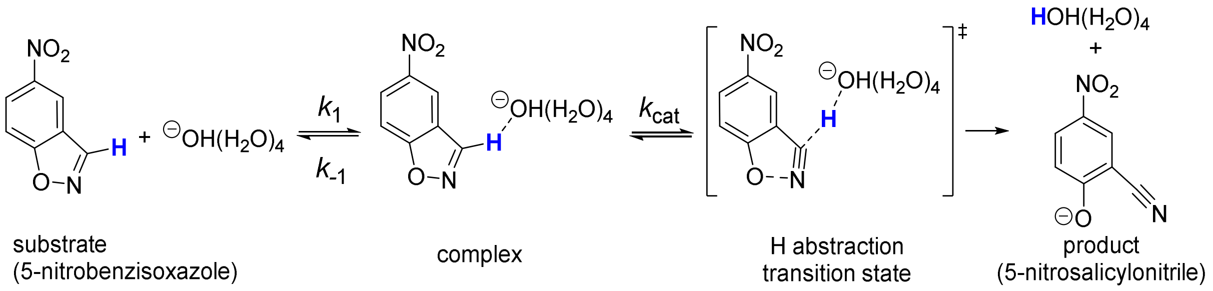

Supplementary Figure 12. Scheme of the investigated small-molecule model reactions.

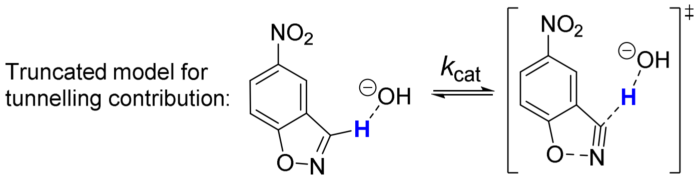

Supplementary Figure 13. Gas phase calculations for the tunneling coefficient at the M06-2X/6-31+G(d) level of theory.

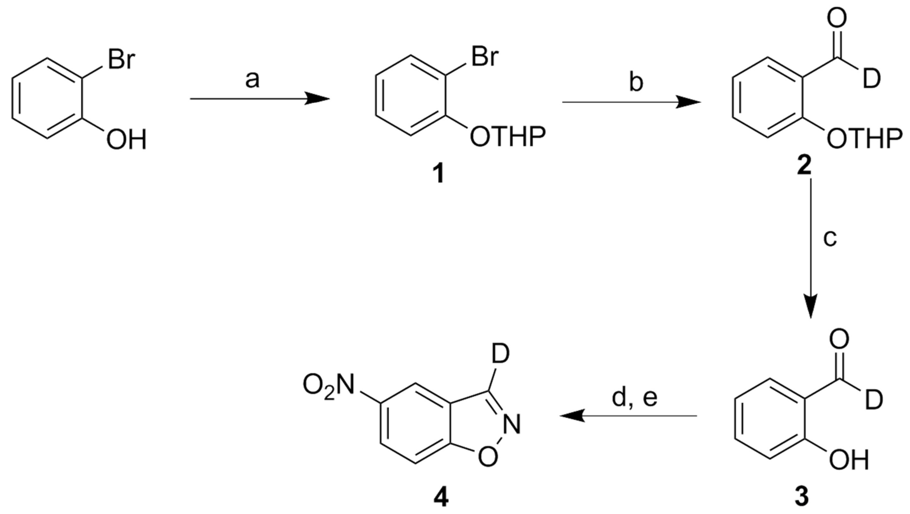

Supplementary Figure 14. Scheme of substrate synthesis. Reagents and conditions: a) 2,3-Dihydropyran, pyridinium p-toluenesulfonate, CH_2Cl_2, 86%; b) 1) 2.2M n-BuLi in hexanes, Et_2O, 0 °C , 2) $[D^7]$-DMF in Et_2O, rt, 94%; c) 1M aq. DCl in D2O, THF, 75%; d) 1) hydroxylamine-O-sulfonic acid, Et_2O, 2) aq. $NaHCO_3$; e) HNO_3/H_2SO_4, 0 °C , 28% over 2 steps.

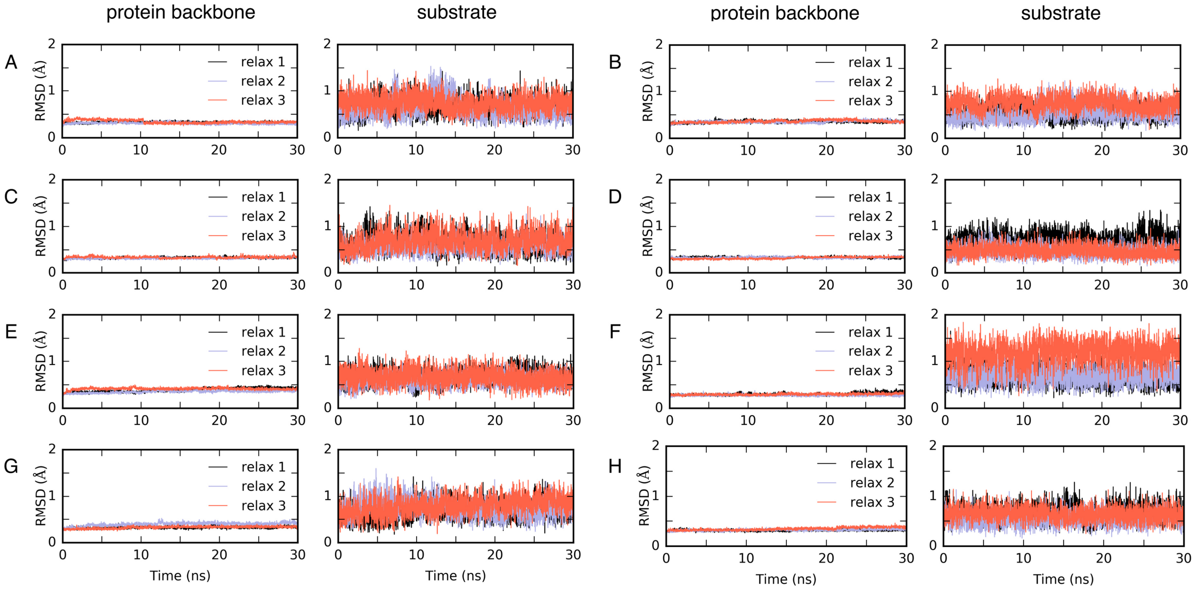

Supplementary Figure 15. Overview of the root mean square deviations (RMSD) of backbone and substrate atoms. Data was taken from the 30 ns equilibrations performed in preparation for empirical valence bond (EVB) simulations of the Kemp elimination of 5-nitrobenzisoxazole, as catalysed by the R1, R5, R7, and R7-2 KE07 variants. The starting crystal structures were (**A**) 4Z08, (**B**) 5D2W, (**C**) 5D30 (lower occupancy of Trp50), (**D**) 5D30 (higher occupancy of Trp50), (**E**) 6DCI chain A, (**F**) 5D33 chain A, (**G**) 5D38 chain A, (**H**) 6CT3. Three independent equilibrations were performed from each starting structure, as described in the Methods section.

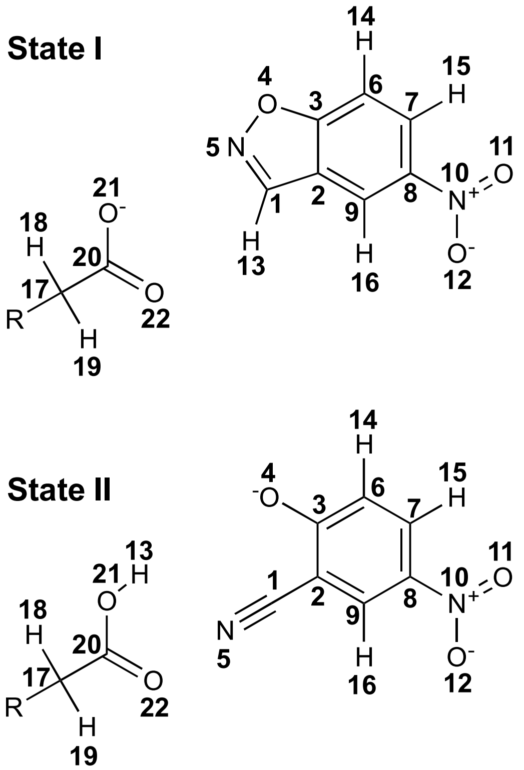

Supplementary Figure 16. Atom numbering used to define the atoms in the different valence bond states. States I and II refer to the Michaelis complex and product states respectively.

Supplementary Tables

Supplementary Table 1. Tryptophan fluorescence parameters

KE07 variants		Temperature (K)					ΔF_{max} [c]	% ΔF_{max} [d]
		283	293	303	313	323	(283 − 323 K)	
R1 (Trp50Ala)	F_{max} [a]	124	124	126	131	133	-9	-8%
	λ_{max} [b]	321	320	320	318	320		
R7-2 (Trp50Ala)	F_{max}	107	106	99	103	101	7	6%
	λ_{max}	320	322	321	322	319		
R1	F_{max}	71	66	63	60	55	16	23%
	λ_{max}	338	334	334	338	338		
R4	F_{max}	132	123	106	99	92	40	30%
	λ_{max}	337	342	338	336	339		
R5	F_{max}	129	121	113	102	89	40	31%
	λ_{max}	340	340	336	338	336		
R6	F_{max}	103	98	91	82	69	35	33%
	λ_{max}	345	347	345	347	337		
R7	F_{max}	35	32	29	27	24	11	32%
	λ_{max}	352	350	348	348	348		
R7-2	F_{max}	29	26	24	22	20	8	29%
	λ_{max}	338	338	336	342	342		

[a] Fluorescence maximum intensity and [b] corresponding wavelength

[c] $\Delta F_{max} = F_{max}$ (283 K) − F_{max} (323 K): Intensity difference between 283 K and 323 K

[d] $\% \Delta F_{max} = \frac{\Delta F_{max}\,(283 - 323\text{ K})}{F_{max}(283\text{ K})} \times 100$

Supplementary Table 2. Accumulation of mutations during KE07 evolution

No.	Resi.	R1	R2	R3	R4	R5	R6	R7	R7-2
1	N224		D	D	D	D	D	D	D
2	G202		R	R	R	R	R	R	R
3	I7			Q	D	D	D	D	D
4	V12					M		L	M
5	K146		T	T	E		T	T	T
6	F77							I	I
7	F229			S				S	S
8	I102								F
9	K19		E				E		
10	Q123		R						
11	F86			L					
12	H84							Y	
13	M207							T	

Mutations throughout the evolution of KE07. Mutations No. 1-3 directly influence the active site electrostatics. Mutations No. 4-8 indirectly optimize the active site preorganization.

Supplementary Table 3. Mutagenesis of Trp50 to Alanine in KE07 variants

	k_{cat} (s^{-1})	K_M (mM)	k_{cat}/K_M ($s^{-1}\cdot M^{-1}$)
R1	0.016 (0.001)	0.76 (0.11)	20.6 (3.5)
R1_Trp50Ala	0.0003 (0.00004) (2.2 %)	0.82 (0.16)	0.42 (0.09) (2 %)
Fold change	45-fold (-)	1	49-fold (-)
R7-2	2.57 (0.12)	0.45 (0.04)	5770 (622)
R7-2_Trp50Ala	0.04 (0.01) (1.7 %)	3.32 (1.32)	13.4 (6.8) (0.2 %)
Fold change	58-fold (-)	7-fold (+)	430-fold (-)

The mean and standard deviations of the kinetic parameters for the mutants were calculated from three independent measurements at 298 K (pH 7.25) with 5-nitrobenzisoxazole. The relative magnitudes of the kinetic parameter for the Trp50Ala mutant compared to the non-mutated variant are given as percentages.

Supplementary Table 4. Comparison of experimental ($\Delta G^{\ddagger}_{exp}$) and calculated (empirical valence bond, $\Delta G^{\ddagger}_{calc}$) activation free energies

	PDB ID: Chain	Trp50 Conformation	$\Delta G^{\ddagger}_{exp}$	$\Delta G^{\ddagger}_{calc}$
R1	4Z08:A	A	20.1	20.2±0.6
	5D2W:A	A		20.5±0.8
R5	5D30:A	A	17.4	20.0±0.8
	5D30:A	B		16.5±0.5
R7	6DCI:A	A	17.1	19.0±0.7
	5D33:A	B		17.8±0.8
R7-2	5D38:A	B	16.9	16.8±0.7
	6CT3: A	C		16.4±1.6

All values are shown in kcal mol^{-1}. $\Delta G^{\ddagger}_{exp}$ was obtained from the kinetic parameters shown in Table 1 of the main text using transition state theory. $\Delta G^{\ddagger}_{calc}$ was obtained from 9 independent EVB simulations for each of the crystal structures and Trp50 conformations, as described in the Methods. For the R1 variant, two structures were used with Trp50 in conformation A. For the R5 variant, the simulations were performed with one structure, in which Trp50 was present in both conformations A (low occupancy) and B (high occupancy). In the case of the R7 and R7-2 variants, the simulation was again performed with two structures, with Trp50 in conformations A and B (R7) or B and C (R7-2). For a definition of the different Trp50 conformations, see the main text.

Supplementary Table 5. Average D-H distances, D-H...A angles, and Glu101 side chain pK_a values at the Michaelis complexes of our empirical valence bond simulations

Variant	Trp50 conformation	D…A	D…H…A	Glu101 pK_a
R1	A	2.8 ± 0.3	153.7 ± 11.0	5.3 ± 0.3
	A	2.7 ± 0.1	160.3 ± 7.8	5.5 ± 0.2
R5	A	2.7 ± 0.1	166.2 ± 6.8	7.6 ± 0.3
	B	2.7 ± 0.2	154.0 ± 9.4	5.7 ± 0.6
R7	A	2.8 ± 0.1	149.5 ± 11.1	6.1 ± 0.3
	B	2.7 ± 0.1	162.2 ± 6.7	7.5 ± 0.2
R7-2	B	3.0 ± 0.3	146.9 ± 12.4	7.4 ± 0.3
	C	2.7 ± 0.2	160.4 ± 10.0	7.5 ± 0.3

All distances are shown in Å, and all angles are shown in °. For each of the crystal structures, 9 independent EVB simulations were performed, as described in the Methods. Glu101 pK_a values are estimated with PROPKA 3.1, using one snapshot every 0.5 ns from the equilibration runs.

Supplementary Table 6. Key reacting distances at the Michaelis complexes and transition states of our empirical valence bond simulations

	Variant	Trp50 conformation	O…H	C…H	N…O
Michaelis complex	R1	A	1.8 ± 0.4	1.1 ± 0.03	1.5 ± 0.03
		A	1.6 ± 0.1	1.1 ± 0.04	1.5 ± 0.03
	R5	A	1.6 ± 0.1	1.1 ± 0.04	1.5 ± 0.03
		B	1.7 ± 0.2	1.1 ± 0.04	1.5 ± 0.03
	R7	A	1.8 ± 0.2	1.1 ± 0.04	1.5 ± 0.03
		B	1.6 ± 0.1	1.1 ± 0.04	1.5 ± 0.03
	R7-2	B	2.0 ± 0.4	1.1 ± 0.04	1.5 ± 0.03
		C	1.6 ± 0.2	1.1 ± 0.04	1.5 ± 0.03
Transition state	R1	A	1.1 ± 0.04	1.6 ± 0.1	2.0 ± 0.1
		A	1.1 ± 0.04	1.6 ± 0.1	2.0 ± 0.1
	R5	A	1.1 ± 0.05	1.6 ± 0.1	2.0 ± 0.1
		B	1.1 ± 0.04	1.6 ± 0.1	2.0 ± 0.1
	R7	A	1.1 ± 0.05	1.6 ± 0.1	2.0 ± 0.1
		B	1.1 ± 0.04	1.6 ± 0.1	2.0 ± 0.1
	R7-2	B	1.1 ± 0.04	1.7 ± 0.1	1.9 ± 0.1
		C	1.1 ± 0.04	1.6 ± 0.1	2.0 ± 0.1

All distances are shown in Å. O..H, C…H, and N…O denote the distances between the oxygen atom of Glu101 and the proton being transferred, the distance between the carbon atom of the substrate and the proton being transferred, and the breaking N-O bond. For each of the crystal structures, 9 independent EVB simulations were performed, as described in the Methods.

Supplementary Table 7. Average stacking distances and angles between the indole ring of Trp50 and the benzisoxazole ring of the substrate during equilibrations in preparation for our empirical valence bond simulations

Variant	Trp50 conformation	Stacking distance	Stacking angle
R1	A	4.4 ± 0.3	20.3 ± 8.2
	A	4.4 ± 0.2	19.7 ± 7.0
R5	A	3.9 ± 0.2	6.9 ± 4.1
	B	3.8 ± 0.2	10.3 ± 4.9
R7	A	4.1 ± 0.2	14.6 ± 9.7
	B	4.1 ± 0.2	14.1 ± 5.3
R7-2	B	4.1 ± 0.3	16.8 ± 8.2
	C	4.2 ± 0.3	11.3 ± 5.0

All distances are shown in Å, and all angles are shown in °. Stacking distance denotes the distance between the centers of mass of the heavy atoms in the corresponding rings. Stacking angle denotes the angle between the normal vectors to the planes of the rings. For each of the crystal structures, 9 independent EVB simulations were performed, as described in the Methods.

Supplementary Table 8. Energies, enthalpies and free energy corrections of all species studied using quantum mechanics at 298 K[a,b,c]

	G_{corr} (H)	H_{corr} (H)	G_{corr} (D)	H_{corr} (D)	E_{elec}	G	H	G (D)	H (D)
					chloroform optimized (298.15 K)				
Substrate	0.074518	0.11842	-	-	-603.9813833	-603.9068653	-603.8629633	-	-
$^{\cdot}OH(H_2O)_4$	0.075545	0.122442	-	-	-381.565889	-381.490344	-381.443447	-	-
complex	0.166548	0.242547	0.16314	0.239385	-985.5552218	-985.3886738	-985.3126748	-985.3920818	-985.3158368
TS	0.164245	0.23876	0.162348	0.236998	-985.5393808	-985.3751358	-985.3006208	-985.3770328	-985.3023828
product + $(H_2O)_5$	0.165885	0.244258	-	-	-985.6271131	-985.4612281	-985.3828551	-	-
					water optimized (298.15 K)				
Substrate	0.074156	0.118096	-	-	-603.977822	-603.903666	-603.859726	-	-
$^{\cdot}OH(H_2O)_4$	0.068436	0.11917	-	-	-381.6029103	-381.5344743	-381.4837403	-	-
complex	0.161104	0.2397	0.157679	0.236522	-985.590044	-985.42894	-985.350344	-985.432365	-985.353522
TS	0.15773	0.235143	0.155865	0.233417	-985.5640452	-985.4063152	-985.3289022	-985.4081802	-985.3306282
product + $(H_2O)_5$	0.162298	0.241618	-	-	-985.659719	-985.497421	-985.418101	-	-
					gas phase optimized (298.15 K)				
Substrate	0.075027	0.118615	-	-	-603.9674226	-603.8923956	-603.8488076	-	-
-OH	-0.007557	0.012004	-	-	-75.75888356	-75.76644056	-75.74687956	-	-
complex	0.081137	0.13118	0.077858	0.128142	-679.7884986	-679.7073616	-679.6573186	-679.7106406	-679.6603566
TS	0.079232	0.128353	0.0766	0.125926	-679.7875278	-679.7082958	-679.6591748	-679.7109278	-679.6616018
product + H_2O	0.078576	0.132769	-	-	-679.9133924	-679.8348164	-679.7806234	-	-

[a] Notations as shown in Supplementary Fig. 13. H and D denote the abstracted isotope – hydrogen or deuterium; [b] Calculated at the M06-2X/6-31+G(d,p) level of theory; [c] All values in Hartrees.

Supplementary Table 9. Calculated tunneling coefficients κ and rate coefficients k for hydrogenated and deuterated versions of the reactions and tunnelling corrected KIEs

T (K)	κ		*k* Pre-complex s^{-1}			*k* Isolated Reactants L mol^{-1} s^{-1}		
	H	D	H	D	KIE	H	D	KIE
283	1.4445	1.2870	6.945E+01	1.226E+01	6.36	6.010E-03	1.064E-03	6.34
293	1.4146	1.2677	1.627E+02	3.034E+01	5.98	1.080E-02	2.022E-03	5.96
298	1.4008	1.2588	2.437E+02	4.669E+01	5.81	1.429E-02	2.747E-03	5.79
303	1.3877	1.2503	3.602E+02	7.079E+01	5.65	1.874E-02	3.691E-03	5.64
313	1.3633	1.2346	7.599E+02	1.565E+02	5.36	3.152E-02	6.512E-03	5.34
323	1.3412	1.2203	1.529E+03	3.300E+02	5.11	5.144E-02	1.112E-02	5.08

The rate coefficients were calculated for the reaction in water both from the pre-complex and from the isolated reactants, the latter for comparison with experiment.

Supplementary Table 10. EVB parameters used in this work[a]

	H_{ij} (kcal mol^{-1})	α_{ij} (kcal mol^{-1})
EVB Parameters	225	-324

[a] H_{ij} is the off-diagonal term in the EVB Hamiltonian, while α_{ij} is the energy difference between the two valence bond states in the background reaction. Both parameters were fitted to reproduce an activation free energy ($\Delta G^{\ddagger}$) of 21.2 kcal mol^{-1}, as described in the Methods section.

Supplementary Table 11. Van der Waals parameters of the reacting atoms, corresponding to the valence bond states[a]

Type	A_i ($kcal^{1/2}\cdot mol^{-1/2}\cdot Å^6$)	B_i ($kcal^{1/2}\cdot mol^{-1/2}\cdot Å^3$)	C_i ($kcal\cdot mol^{-1}$)	α_i ($Å^2$)	$A_{1\text{-}4}$ ($kcal^{1/2}\cdot mol^{-1/2}\cdot Å^3$)	$B_{1\text{-}4}$ ($kcal^{1/2}\cdot mol^{-1/2}\cdot Å^3$)	mass (a.u.)
CT	944.52	22.03	1	2.5	667.88	15.58	12.01
CA	1059.13	23.67	1	2.5	748.92	16.74	12.01
C2	1802.24	34.18	1	2.5	1274.38	24.17	12.01
O2	616.44	23.77	90	2.5	435.89	16.81	16.00
O1	616.44	23.77	1	2.5	435.89	16.81	16.00
ODE	601.15	22.27	90	2.5	425.08	15.74	16.00
CR1	1059.13	23.67	180	2.6	748.92	16.74	12.01
CR2	1831.61	37.67	180	2.6	1295.14	26.63	12.01
N1	971.75	28.31	180	2.6	687.13	20.02	14.01
N2	885.43	27.02	180	2.6	626.09	19.11	14.01
NO	816.43	23.78	1	2.5	577.31	16.82	14.01
ON	554.63	21.39	1	2.5	392.18	15.12	16.00
OS	445.13	18.25	180	2.6	314.75	12.91	16.00
OR	616.44	23.77	180	2.6	435.89	16.81	16.00
HA	69.58	4.91	5	2.5	49.20	3.47	1.01
HC	84.57	5.41	1	2.5	59.80	3.83	1.01
HO	0.00	0.00	5	2.5	0.00	0.00	1.01

[a] A standard 6-12 Lennard Jones potential was used for all atoms except reacting atoms. In the case of the reacting atoms, which changed bonding patterns between atoms *i* and *j*, an alternate function of the form: $V_{react} = C_i C_j \exp(-\alpha_i \alpha_j r_{ij})$ was used in order to prevent artificial repulsion between the reacting atoms as their bonding patterns changed during the course of the reaction. r_{ij} denotes the distance (in Å) between atoms *i* and *j*.

Supplementary Table 12. Atom types of the reacting atoms corresponding to the valence bond states[a]

Atom Number	State I	State II
1	CR1	CR2
2	CA	CA
3	CA	CA
4	OS	OR
5	N1	N2
6	CA	CA
7	CA	CA
8	CA	CA
9	CA	CA
10	NO	NO
11	ON	ON
12	ON	ON
13	HA	HO
14	HA	HA
15	HA	HA
16	HA	HA
17	CT	CT
18	HC	HC
19	HC	HC
20	C2	C2
21	O2	ODE
22	O2	O1

Supplementary Table 13. Partial charges of the reacting atoms, corresponding to the valence bond states

#	State I	State II
1	0.1634	0.4584
2	-0.0516	-0.3286
3	0.5146	0.7236
4	-0.1794	-0.6838
5	-0.3257	-0.5876
6	-0.4238	-0.4576
7	-0.0613	-0.0906
8	-0.0588	-0.1376
9	-0.2382	-0.1061
10	0.8385	0.7998
11	-0.4782	-0.5444
12	-0.4782	-0.5444
13	0.1405	0.4500
14	0.2264	0.1812
15	0.1900	0.1598
16	0.2218	0.1579
17	-0.2200	-0.1200
18	0.0600	0.0600
19	0.0600	0.0600
20	0.7000	0.5200
21	-0.8000	-0.5300
22	-0.8000	-0.4400

Supplementary Table 14. Bond parameters for the covalent bonds of the reacting system[a]

Bond Type	E_D (kcal·mol^{-1})	α (Å^{-2})	r_0 (Å)	k_b (kcal·mol^{-1}·Å^{-2})	b (Å)
0			Not Set		
1				938	1.4000
2				938	1.4210
3				800	1.4510
4				900	1.3640
5				820	1.3200
6				1300	1.1570
7				734	1.0800
8				800	1.4600
9				1100	1.2250
10				536	1.529
11				634	1.522
12				680	1.090
13				1312	1.250
14				1140	1.229
15				900	1.364
16	138.25	2	0.9450		
17	91.75	2	1.0800		
18	295.68	1.25	1.3990		

[a] Morse bond (reacting atoms): $V_{Morse} = D_e\{1-exp[-\alpha (r_{ij}-r_0)]\}^2$. Harmonic bonds (non-reacting atoms): $V_{harmonic} = 0.5k (r_{ij}-r_0)^2$.

Supplementary Table 15. Bond types between the reacting atoms, corresponding to the valence bond states

Number		Bond Type	
#1	#2	State I	State II
1	2	2	3
2	3	1	1
3	4	4	4
4	5	18	0
1	5	5	6
3	6	1	1
6	7	1	1
7	8	1	1
8	9	1	1
2	9	1	1
8	10	8	8
10	11	9	9
10	12	9	9
1	13	16	0
6	14	7	7
7	15	7	7
9	16	7	7
17	18	12	12
17	19	12	12
17	20	11	11
17	R	10	10
20	21	13	15
20	22	13	14
21	13	0	17

Supplementary Table 16. Angle parameters used for bending adjacent bonds in the reacting system[a]

Angle Type	k_a (kcal·mol^{-1}·rad^{-2})	Θ_0 (°)
0	No Set	
1	70	128.6
2	70	118.9
3	140	111.9
4	126	120.0
5	300	180.0
6	140	120.0
7	140	105.3
8	140	108.9
9	70	120.0
10	170	120.0
11	160	117.5
12	160	125.3
13	75	110.7
14	66	107.8
15	70	109.5
16	126	111.1
17	140	117.0
18	160	120.4
19	160	126.0
20	70	113.0

[a] Angle potential: $V_{angle}=0.5\sum k\,(\Theta - \Theta_0)^2$.

Supplementary Table 17. Angle types between the reacting atoms, corresponding to the valence bond states

Atom number			Angle Type	
#1	#2	#3	State I	State II
1	2	3	4	6
1	2	9	4	6
13	1	2	1	0
13	1	5	2	0
5	1	2	3	0
2	3	4	6	6
2	3	6	4	4
3	4	5	8	0
3	6	7	4	4
3	6	14	9	9
6	7	8	4	4
6	7	15	9	9
7	8	9	4	4
7	8	10	10	10
2	9	8	4	4
8	10	11	11	11
8	10	12	11	11
11	10	12	12	12
8	9	16	9	9
R	17	18	13	13
R	17	19	13	13
R	17	20	16	16
18	17	20	15	15
19	17	20	15	15
17	20	21	17	17
17	20	22	17	18
21	20	22	19	19
20	21	13	0	20

Supplementary Table 18. Torsion parameters used to describe the reacting system[a]

Torsion Type	V_1	V_2	V_3	Torsion Type	V_1	V_2	V_1
	0.5·barrier height (kcal·mol^{-1})				0.5·barrier height (kcal·mol^{-1})		
0		Not Set		3	0.0000	2.4500	0.0000
1	0.0000	3.6250	0.0000	4	1.5000	2.4500	0.0000
2	0.0000	0.2730	0.0000				

[a] Torsion angle potential: $V_{torsion}= V_1 (1+\cos(n\varphi -\delta))+ V_2 (1+\cos2(n\varphi -\delta))+ V_3 (1+\cos3(n\varphi -\delta))$, *n* is the periodicity (number of maxima per turn) and δ is the phase shift.

Supplementary Table 19. Torsion types between the reacting atoms, corresponding to the valence bond states

Atom number				Torsion type	
#1	#2	#3	#4	State I	State II
13	1	2	3	1	0
13	1	2	9	1	0
13	1	5	4	1	0
1	2	9	16	1	1
1	2	3	6	1	1
1	2	3	4	1	1
1	5	4	3	1	0
1	2	9	8	1	1
2	3	6	7	1	1
2	3	6	14	1	1
2	3	4	5	1	0
2	9	8	7	1	1
2	9	8	10	1	1
3	6	7	8	1	1
3	6	7	15	1	1
4	3	2	9	1	1
4	3	6	14	1	1
4	3	6	7	1	1
4	5	1	2	1	0
5	1	2	3	1	0
5	1	2	9	1	0
6	7	8	9	1	1
6	7	8	10	1	1
4	3	6	14	1	1
14	6	7	15	1	1
14	6	7	8	1	1
7	8	9	16	1	1
7	8	10	11	1	1
7	8	10	12	1	1
15	7	8	10	1	1
16	9	8	10	1	1
9	8	10	11	1	1
9	8	10	12	1	1
8	9	2	3	1	1
9	2	3	6	1	1
R	17	20	21	2	2
R	17	20	22	2	2
18	17	20	21	0	0
18	17	20	22	0	0
19	17	20	21	0	0
19	17	20	22	0	0
17	20	21	13	0	4
22	20	21	13	0	3

Supplementary Table 20. Improper torsion parameters used to describe the reacting system[a]

Improper Type	k_a (kcal·mol^{-1} rad^{-2})	τ_0 (°)
0	Not Set	
1	1.1	180
2	10.5	180

[a] Improper torsion potential: $V_{torsion}=k\,(\tau - \tau_0)^2$. k_i is the force constant and τ is the equilibrium angle (in degrees).

Supplementary Table 21. Improper torsion types between the reacting atoms, corresponding to the valence bond states

Atom Number				Improper Type	
#1	#2	#3	#4	State I	State II
5	1	2	13	1	0
1	2	3	9	1	1
2	3	4	6	1	1
3	6	7	14	1	1
6	7	8	15	1	1
7	8	9	10	1	1
8	10	11	12	2	2
2	9	8	16	1	1
17	20	21	22	1	1

Supplementary Notes

Supplementary Note 1. Geometries for small-molecule study

Geometries of all species, optimized at the M06-2X/6-31+G(d,p) level of theory, in the form of Gaussian archive entries.

Water optimised

Substrate

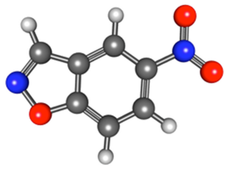

```
1\1\GINC-R255\FOpt\RM062X\6-31+G(d,p)\C7H4N2O3\ROOT\04-Oct-2016\0\\# m
 062x/6-31+g(d,p) opt=(maxcyc=200) freq=noraman int=ultrafine scf=maxcy
 c=200 # scrf=(smd,solvent=water)\\Oxazole\\0,1\C,-1.1133761167,0.22476
 00961,0.\C,-1.0637696931,-1.1837104302,0.\C,0.1541832272,-1.8358123199
 ,0.\C,1.2836217119,-1.0153279039,0.\C,1.2321982264,0.3787319676,0.\C,0
 .0044661345,1.0409788144,0.\N,-2.4283115145,0.8637840162,0.\H,-1.98726
 59735,-1.7493292942,0.\H,0.2289010907,-2.9163403811,0.\O,2.5712809873,
 -1.4176234964,0.\C,2.6173591152,0.7578279714,0.\H,-0.0745705164,2.1212
 035531,0.\N,3.3918925168,-0.2821469574,0.\O,-2.4806688101,2.0853592232
 ,0.\O,-3.421052556,0.1503087415,0.\H,3.0514771703,1.7486513997,0.\\Ver
 sion=ES64L-G09RevE.01\State=1-A'\HF=-603.977822\RMSD=7.482e-09\RMSF=8.
 558e-05\Dipole=0.8896047,-0.2083057,0.\Quadrupole=-15.43453,10.0832337
 ,5.3512964,12.358003,0.,0.\PG=CS [SG(C7H4N2O3)]\\@
```

$^{-}$OH

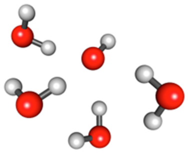

```
1\1\GINC-R262\FOpt\RM062X\6-31+G(d,p)\H9O5(1-)\ROOT\04-Oct-2016\0\\# m
 062x/6-31+g(d,p) opt=(maxcyc=200) freq=noraman int=ultrafine scf=maxcy
 c=200 # scrf=(smd,solvent=water)\\OH(-)+4 H2O complex\\-1,1\O,-1.31764
 50138,-0.1216758034,-1.1032174162\H,-1.7755235562,-0.7972072116,-1.616
 389323\H,-0.9195706672,-0.2558151975,0.5027123491\O,-0.5904846622,-0.1
 330439553,1.4358604316\H,-0.1963281526,0.753503965,1.4084045797\H,0.70
 08781283,1.8812242563,-0.311486554\O,0.28279763,2.4763571065,0.3435654
 313\H,-0.6554514434,2.5038632758,0.0644108596\H,-2.4798125149,2.626843
 1192,-1.4147412797\O,-2.3476822986,2.1276056187,-0.5993686822\H,-2.005
 8704266,1.1968010617,-0.8791287404\H,1.6563819109,-0.1009783941,-1.138
 1482882\O,1.0766543518,0.5444674714,-1.5616368701\H,0.1166567146,0.194
 0546871,-1.4358364974\\Version=ES64L-G09RevE.01\State=1-A\HF=-381.6029
 103\RMSD=6.668e-09\RMSF=1.632e-05\Dipole=-0.0263058,-0.2701488,-1.6607
 965\Quadrupole=1.4669642,1.4650964,-2.9320606,-3.002852,5.1925275,1.23
 4555\PG=C01 [X(H9O5)]\\@
```

complex

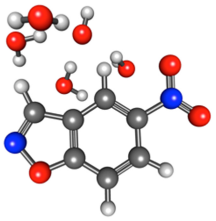

```
1\1\GINC-R192\FOpt\RM062X\6-31+G(d,p)\C7H13N2O8(1-)\ROOT\06-Oct-2016\0
 \\# m062x/6-31+g(d,p) opt=(maxcyc=200) freq=noraman int=ultrafine scf=
 maxcyc=200 # scrf=(smd,solvent=water) iop(1/8=1)\\OH+(H2O)4---oxazole
 complex (SMD opt)\\-1,1\C,-1.7167754232,0.0691003139,0.3485610842\C,-2
 .102827116,0.9982086993,-0.6379636286\C,-1.2472555775,2.0218055504,-0.
 9996107667\C,-0.0160954751,2.0461620078,-0.3411110412\C,0.3644046864,1
 .1228640877,0.6328426509\C,-0.5003134688,0.0934907322,1.0063628588\N,-
 2.6546805753,-0.9958483674,0.6903615123\H,-3.0761359864,0.9058042614,-
 1.1043734786\H,-1.5123353943,2.7555738289,-1.7509981494\O,0.9831883409
 ,2.9302700991,-0.5390021897\C,1.6884643228,1.5498736453,0.9895793735\H
 ,-0.2326893757,-0.6490660002,1.7484502801\N,2.0449202007,2.598303358,0
 .3133884945\O,-2.4159764244,-1.6885664799,1.6676907649\O,-3.6377779066
 ,-1.1528617611,-0.0190845864\H,2.3683118611,1.1277379059,1.7178142256\
 O,1.5973580673,-1.9443893995,-0.3912342638\H,2.0108519851,-2.753907878
 7,-0.722000724\H,2.8967858606,-1.1426563092,2.1815509722\O,2.112322532
 7,-1.704657069,2.1515475568\H,1.9047878157,-1.8127199879,1.1701032341\
 H,0.006113816,-2.3821131824,-0.6223763067\O,-0.9385247951,-2.65529876,
 -0.803490572\H,-0.8732821088,-3.3557413914,-1.4644893129\H,2.968810078
 1,-0.9543066587,-0.4471429559\O,3.8077653445,-0.4197338028,-0.47874894
 87\H,3.5526491708,0.4244808607,-0.8719718451\H,1.2238197212,-0.9425977
 974,-1.6211902129\O,1.0133090642,-0.3053590376,-2.366396423\H,0.067361
 7591,-0.1252214676,-2.2853636019\\Version=ES64L-G09RevE.01\State=1-A\H
 F=-985.590044\RMSD=8.764e-09\RMSF=9.889e-06\Dipole=-2.0276616,2.936470
 7,-0.4195572\Quadrupole=-13.34689,6.1690868,7.1778032,-11.015716,9.564
 6399,9.0713024\PG=C01 [X(C7H13N2O8)]\\@
```

TS

```
1\1\GINC-R172\FTS\RM062X\6-31+G(d,p)\C7H13N2O8(1-)\ROOT\05-Oct-2016\0\
```

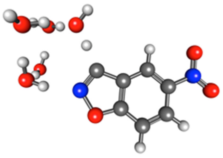

\# m062x/6-31+g(d,p) opt=(TS,calcfc,noeigen,maxcyc=200,cartesian) freq
=noraman int=ultrafine scf=maxcyc=200 # scrf=(smd,solvent=water) iop(1
/8=1)\\TS for H abstraction by water network (SMD opt)\\-1,1\C,2.65010
15427,-0.0261748559,-0.1449528201\C,2.9470863105,1.3494825324,-0.11472
20649\C,1.9555498841,2.2796807254,-0.3654328498\C,0.6789611297,1.77488
38492,-0.6390047776\C,0.388711251,0.4034834547,-0.6712514718\C,1.38544
28486,-0.5325766366,-0.4182897742\N,3.7258495616,-0.9664620455,0.12434
28258\H,3.9572579206,1.6722258012,0.1061403796\H,2.1578803617,3.343943
2704,-0.3477418298\O,-0.4186016357,2.4841758941,-0.9015105779\C,-1.028
9124945,0.3060687344,-0.9819831767\H,1.1910684736,-1.5989154629,-0.432
9908034\N,-1.5102304484,1.4964233719,-1.1225574928\O,3.4779015371,-2.1
657710811,0.0933365008\O,4.8419367872,-0.5261166443,0.3723611032\H,-1.
8502799438,-0.8545043347,-1.034205826\O,-4.8311615418,-1.0584479805,0.
0580375512\H,-5.487129827,-0.9467637392,-0.6416926968\H,-2.4154280148,
-2.3706512418,-1.717788984\O,-2.5133914947,-1.8265699129,-0.9252928286
\H,-4.0067248711,-1.4014659618,-0.387610796\H,-1.6390233871,-2.8405093
251,1.9289007755\O,-1.1379970586,-2.2717202523,1.3303004687\H,-1.66382
54301,-2.2340425316,0.4914304445\H,-4.1819036909,0.5481322004,0.542883
5449\O,-3.7420504816,1.3820753682,0.8164695462\H,-3.1554855005,1.61141
76187,0.0751610744\H,-2.3037164026,0.7050014959,1.8348825232\O,-1.5365
838271,0.3568152929,2.3275350844\H,-1.3784465576,-0.5352826035,1.96134
4948\\Version=ES64L-G09RevE.01\State=1-A\HF=-985.5640452\RMSD=4.662e-0
9\RMSF=3.494e-06\Dipole=1.3069427,-0.5025461,-2.6536177\Quadrupole=-17
.7123747,10.2338486,7.4785261,22.7340133,9.7361131,1.2186085\PG=C01 [X
(C7H13N2O8)]\\@

product

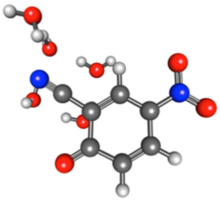

1\1\GINC-R254\FOpt\RM062X\6-31+G(d,p)\C7H13N2O8(1-)\ROOT\04-Oct-2016\0
\\# m062x/6-31+g(d,p) opt=(maxcyc=200) freq=noraman int=ultrafine scf=
maxcyc=200 scrf=(smd,solvent=water)\\Product IRC opt (SMD opt)\\-1,1\C
,2.453419518,-0.1455807518,0.1401723038\C,2.6449270331,1.2427282002,0.
3219921997\C,1.6929155052,2.1265509162,-0.1089903595\C,0.477909427,1.6
858127395,-0.7399322822\C,0.3473677569,0.2555891087,-0.9149080422\C,1.
3120577981,-0.6419622109,-0.4801939257\N,3.448014009,-1.0597625666,0.5
972662119\H,3.5475442661,1.5977615728,0.8063305795\H,1.8266710258,3.19
42695266,0.0307698122\O,-0.4334427088,2.490495088,-1.1073516549\C,-0.8
443292822,-0.2239909715,-1.5362730292\H,1.1730287312,-1.708316756,-0.6
175635949\N,-1.8147433493,-0.5926191296,-2.0565631294\O,3.277399391,-2
.2696008944,0.4299671426\O,4.4562123417,-0.6151167742,1.150186796\H,-3
.689501891,-1.2417706664,-2.1803142532\O,-3.6691589115,-0.7052879832,0
.6531617351\H,-4.3009154362,-0.8030063113,1.3778662346\H,-5.2082780196
,-0.9664317038,-2.2482095323\O,-4.5492953102,-1.5396459664,-1.83491416
5\H,-4.1277290574,-1.0081654404,-0.1592041266\H,-2.1158186564,-1.59692
9742,0.951795206\O,-1.2288573091,-1.9723740925,1.1325685717\H,-1.37184
94235,-2.6536454514,1.8026572546\H,-2.8275197254,0.9507238377,0.609779
4693\O,-2.3583046954,1.7795917942,0.8257632921\H,-1.8554705824,2.03748
59921,0.0303424955\H,-1.0007750899,1.0213182982,1.8909924277\O,-0.3777
422763,0.451854664,2.3827181722\H,-0.5169810788,-0.4285853259,1.986015
1908\\Version=ES64L-G09RevE.01\State=1-A\HF=-985.659719\RMSD=6.955e-09
\RMSF=1.381e-05\Dipole=-2.8920555,-1.4848365,-0.6235837\Quadrupole=4.3
780821,-6.0915498,1.7134677,17.096965,-9.0814406,-3.6572083\PG=C01 [X(
C7H13N2O8)]\\@

Chloroform optimised

Substrate

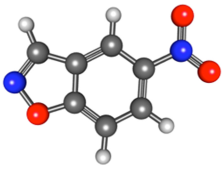

1\1\GINC-R237\FOpt\RM062X\6-31+G(d,p)\C7H4N2O3\ROOT\30-Sep-2016\0\\# m
062x/6-31+g(d,p) opt=(maxcyc=200) freq=noraman int=ultrafine scf=maxcy
c=200 # scrf=(smd,solvent=chloroform)\\Oxazole\\0,1\C,-1.1152963767,0.
2250858154,0.\C,-1.0626820523,-1.1828007721,0.\C,0.1566766755,-1.83338
01339,0.\C,1.2876972318,-1.0135634171,0.\C,1.23303749,0.3813609346,0.\
C,0.0021129198,1.0404601461,0.\N,-2.4356405971,0.8656984474,0.\H,-1.98
78004951,-1.7463225345,0.\H,0.2326678435,-2.9141665406,0.\O,2.56959513
91,-1.4151847457,0.\C,2.6210157986,0.7564169948,0.\H,-0.0863714208,2.1
200602099,0.\N,3.3892034503,-0.2880604739,0.\O,-2.4806820754,2.0851748
062,0.\O,-3.4213669429,0.1468577305,0.\H,3.0641984117,1.743678533,0.\\
Version=ES64L-G09RevE.01\State=1-A'\HF=-603.9813833\RMSD=7.544e-09\RMS
F=7.391e-05\Dipole=0.769832,-0.2063233,0.\Quadrupole=-13.8136336,9.211
0417,4.6025919,11.082224,0.,0.\PG=CS [SG(C7H4N2O3)]\\@

$^{-}$OH

1\1\GINC-R333\FOpt\RM062X\6-31+G(d,p)\H9O5(1-)\ROOT\29-Sep-2016\0\\# m

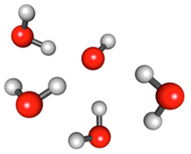

062x/6-31+g(d,p) opt=(maxcyc=200) freq=noraman int=ultrafine scf=maxcy
c=200 # scrf=(smd,solvent=chloroform)\\OH(-)+4 H2O complex\\-1,1\O,-1.
3422762708,-0.1195585086,-1.0640080995\H,-1.8116575799,-0.7953813389,-
1.5635738673\H,-0.8747647562,-0.324771894,0.5907759675\O,-0.4872049418
,-0.2060912652,1.4928071799\H,-0.154561515,0.7045197036,1.4544265842\H
,0.6646111269,1.9074357761,-0.2880625841\O,0.2410479927,2.4372057803,0
.4167810846\H,-0.6844913502,2.5206074773,0.1106301081\H,-2.3684942398,
2.6183771457,-1.5775988889\O,-2.3092355945,2.1768042783,-0.7228728071\
H,-2.0031432891,1.2053694053,-0.9147907264\H,1.609402491,-0.0560949635
,-1.2074911298\O,1.0108809088,0.6013656152,-1.5806527931\H,0.054887017
9,0.2262127883,-1.4513700281\\Version=ES64L-G09RevE.01\State=1-A\HF=-3
81.565889\RMSD=6.722e-09\RMSF=2.719e-05\Dipole=-0.0527431,-0.2433094,-
1.5250622\Quadrupole=1.3709954,1.1443303,-2.5153257,-1.7292443,3.58419
07,1.6294507\PG=C01 [X(H9O5)]\\@

complex

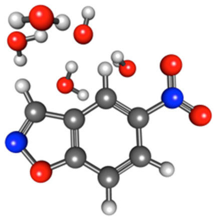

1\1\GINC-R1201\FOpt\RM062X\6-31+G(d,p)\C7H13N2O8(1-)\ROOT\23-Sep-2016\
0\\# m062x/6-31+g(d,p) opt=(maxcyc=200) freq=noraman int=ultrafine scf
=maxcyc=200 scrf=(smd,solvent=chloroform)\\OH+(H2O)4---oxazole complex
 (SMD opt)\\-1,1\C,-1.8027546282,0.0703917022,0.3700059931\C,-2.184412
2332,1.0419437221,-0.5745537453\C,-1.3026273471,2.0424476596,-0.940220
7179\C,-0.042787707,1.9931035354,-0.3400838823\C,0.3296843378,1.028039
4457,0.5957066635\C,-0.5626446254,0.0272004008,0.9795209929\N,-2.75573
31561,-0.9926894136,0.6844568502\H,-3.1742006895,0.9907840201,-1.01213
20189\H,-1.5624849107,2.8016343474,-1.6682575808\O,0.990295312,2.81719
07,-0.5781593404\C,1.6990650781,1.3590254387,0.8747367504\H,-0.2878068
805,-0.7565827959,1.6761563246\N,2.0792804861,2.3954864719,0.192573127
7\O,-2.5444628174,-1.6841749972,1.6657105341\O,-3.7273550825,-1.127973
384,-0.0452877111\H,2.386848345,0.844599918,1.5333481844\O,1.810367132
7,-1.832142053,-0.3540346872\H,2.228677338,-2.6611587298,-0.6212665431
\H,2.6746292822,-1.8389011924,2.5529293773\O,1.8855619993,-1.418842125
7,2.1934191737\H,1.8782051042,-1.6336231246,1.2001370797\H,0.189728021
4,-2.1905298026,-0.6230178614\O,-0.7638036567,-2.335435598,-0.88676214
69\H,-0.8269137532,-3.2575503413,-1.159915864\H,3.1786380624,-0.807383
5799,-0.5422979715\O,3.9941296726,-0.2595161437,-0.6806980681\H,3.6787
006274,0.5869456595,-1.0189949241\H,1.3293091871,-0.8079817665,-1.6629
888163\O,0.9093319227,-0.232386841,-2.3487703368\H,-0.0179114214,-0.50
32901321,-2.3095548355\\Version=ES64L-G09RevE.01\State=1-A\HF=-985.555
2218\RMSD=4.550e-09\RMSF=1.521e-05\Dipole=-1.9613729,0.939643,0.300655
8\Quadrupole=-16.9075234,12.2575668,4.6499567,-13.5847418,11.0683247,2
.6542833\PG=C01 [X(C7H13N2O8)]\\@

TS

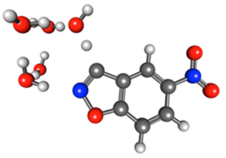

1\1\GINC-R491\FTS\RM062X\6-31+G(d,p)\C7H13N2O8(1-)\ROOT\27-Sep-2016\0\
\# m062x/6-31+g(d,p) opt=(TS,calcfc,noeigen,maxcyc=200) freq=noraman i
nt=ultrafine scf=maxcyc=200 # scrf=(smd,solvent=chloroform) iop(1/8=1)
\\TS for H abstraction by water network (SMD opt)\\-1,1\C,3.284373958,
0.2388038992,-0.8164953344\C,3.4141228643,1.6380429895,-0.7398256493\C
,2.2981394495,2.4285642466,-0.5352228419\C,1.071668971,1.7641415454,-0
.4130742807\C,0.9449037208,0.369146174,-0.4877431844\C,2.0698268071,-0
.4240526703,-0.6937662838\N,4.4895142772,-0.5540236144,-1.0363574075\H
,4.3958070252,2.0845020418,-0.8428496858\H,2.370413237,3.5081233571,-0
.4711007952\O,-0.117042438,2.322519477,-0.2158180235\C,-0.4750311706,0
.0982865929,-0.3089364991\H,2.0122312402,-1.5047230579,-0.7550353036\N
,-1.0856712397,1.2282336049,-0.1554473204\O,4.3780605896,-1.7690235316
,-1.1153755616\O,5.5594075652,0.0312377143,-1.1335967754\H,-1.22839888
47,-1.0841404691,-0.276957467\O,-4.3972375827,-1.2659683115,-0.6355575
756\H,-4.7123605962,-1.2224257121,-1.5455626172\H,-1.5417191669,-2.804
20525,-0.6401978975\O,-1.956857211,-2.0383846022,-0.2247236996\H,-3.45
43389213,-1.6078670355,-0.6520978576\H,-4.3727246527,-2.1811524046,1.4
251852976\O,-3.5796120626,-2.3340494069,1.9585263682\H,-2.8546904112,-
2.289508802,1.2951381824\H,-4.1365199485,0.3624742355,0.2140430781\O,-
3.8671244462,1.1686364623,0.6949661147\H,-2.9505839869,1.3392727643,0.
4090331562\H,-3.7592799606,0.6508607094,2.5118166797\O,-3.6789765391,0
.1616075514,3.3509471993\H,-3.5634454859,-0.7570924975,3.0521829848\\V
ersion=ES64L-G09RevE.01\State=1-A\HF=-985.5393808\RMSD=9.260e-09\RMSF=
5.338e-06\Dipole=3.4317778,0.4186692,-3.5223066\Quadrupole=-31.2010407
,23.2274684,7.9735723,11.7737404,34.2607143,2.5677775\PG=C01 [X(C7H13N
2O8)]\\@

product

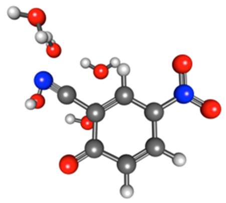

1\1\GINC-R452\FOpt\RM062X\6-31+G(d,p)\C7H13N2O8(1-)\ROOT\30-Sep-2016\0
\\# m062x/6-31+g(d,p) opt=(maxcyc=200) freq=noraman int=ultrafine scf=
maxcyc=200 scrf=(smd,solvent=chloroform)\\Product IRC opt (SMD opt)\\-
1,1\C,2.4836810599,-0.146367978,0.1500449808\C,2.6916559178,1.24906464
75,0.2586672129\C,1.7296354875,2.1229729255,-0.1662619442\C,0.47433938
37,1.6718404238,-0.7196368188\C,0.3378467952,0.2308991649,-0.838006225
3\C,1.3108744589,-0.6548129716,-0.3954002212\N,3.4928401032,-1.0514238
476,0.6050238118\H,3.6185331139,1.6100481605,0.6905445645\H,1.87222080
58,3.1948005717,-0.0725812459\O,-0.4444161081,2.4611639068,-1.06380188
8\C,-0.8726585532,-0.2633159664,-1.4063671679\H,1.1531796139,-1.724761
7241,-0.4637554577\N,-1.8616180742,-0.63548494,-1.8859684278\O,3.29797
07159,-2.2630140038,0.5047222196\O,4.5299864324,-0.5878929936,1.079823
2155\H,-3.6965414317,-1.145815096,-2.1587501349\O,-3.7178208163,-0.852
5341637,0.6580338465\H,-4.3607012361,-0.8846742354,1.377962023\H,-5.18
4767855,-0.7269641537,-2.3163686324\O,-4.6047576784,-1.3575408192,-1.8
711555613\H,-4.1946077401,-1.0652783358,-0.1736174767\H,-2.0652600484,
-1.6677728703,0.8966601554\O,-1.1518719239,-1.9527818874,1.0912069992\
H,-1.2291143316,-2.7052563174,1.6905602109\H,-2.8651021685,0.876962959
5,0.5104652083\O,-2.4186600151,1.7074263289,0.7467640187\H,-1.88978406
76,1.9942267986,-0.0235465179\H,-1.0956760832,1.0592060702,1.950064240
2\O,-0.4743824851,0.5141941244,2.4659861497\H,-0.4982692719,-0.3457247
785,2.0146158629\\Version=ES64L-G09RevE.01\State=1-A\HF=-985.6271131\R
MSD=4.964e-09\RMSF=1.684e-05\Dipole=-2.4144021,-1.2644299,-0.7574186\Q
uadrupole=-0.4035465,-2.0929958,2.4965423,14.1125951,-7.1259052,-3.905
0138\PG=C01 [X(C7H13N2O8)]\\@

Gas phase

Substrate

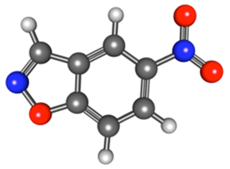

1\1\GINC-R2965\FOpt\RM062X\6-31+G(d,p)\C7H4N2O3\ROOT\10-Oct-2016\0\\#
m062x/6-31+G** opt=(maxcyc=200) scf=maxcyc=200 freq=noraman int=ultraf
ine\\oxazole\\0,1\C,-1.4889779602,2.1481273114,0.\C,-2.6750134208,1.39
02896935,0.\C,-2.617918621,0.0090299951,0.\C,-1.3395875067,-0.55458107
4,0.\C,-0.1641460211,0.1996248316,0.\C,-0.2221233712,1.5954033819,0.\N
,-1.6051015038,3.6161741097,0.\H,-3.624015922,1.9121921055,0.\H,-3.510
2061884,-0.6047111113,0.\O,-1.038781965,-1.8628339994,0.\C,0.857132741
1,-0.8133125334,0.\H,0.6564962389,2.2289655449,0.\N,0.3433260707,-2.00
40793423,0.\O,-0.5727451323,4.2626972094,0.\O,-2.7257052593,4.09277244
76,0.\H,1.9330978211,-0.7046785703,0.\\Version=ES64L-G09RevE.01\State=
1-A'\HF=-603.9674226\RMSD=4.172e-09\RMSF=4.951e-05\Dipole=0.1568552,-0
.6908783,0.\Quadrupole=10.905331,-14.7378312,3.8325002,3.8336358,0.,0.
\PG=CS [SG(C7H4N2O3)]\\@

$^{-}$OH

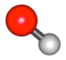

1\1\GINC-R955\FOpt\RM062X\6-31+G(d,p)\H1O1(1-)\ROOT\10-Oct-2016\0\\# m
062x/6-31+G** opt=(maxcyc=200) scf=maxcyc=200 freq=noraman int=ultrafi
ne\\OH anion\\-1,1\O,-0.4617542619,-0.1430832,-0.01426499\H,0.50504578
19,-0.1430832,-0.01426499\\Version=ES64L-G09RevE.01\State=1-SG\HF=-75.
7588836\RMSD=5.310e-09\RMSF=8.074e-05\Dipole=0.7364837,0.,0.\Quadrupol
e=1.1816848,-0.5908424,-0.5908424,0.,0.,0.\PG=C*V [C*(H1O1)]\\@

complex

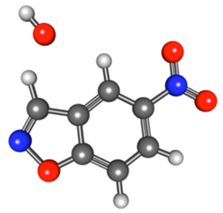

1\1\GINC-R886\FOpt\RM062X\6-31+G(d,p)\C7H5N2O4(1-)\ROOT\10-Oct-2016\0\
\# m062x/6-31+G** opt=(maxcyc=200) scf=maxcyc=200 freq=noraman int=ult
rafine\\Oxazole-OH complex\\-1,1\C,0.998855187,0.3031141274,-0.0170675
075\C,1.022251876,-1.1088971871,-0.0133085283\C,-0.1557159674,-1.83330
9091,0.0030545005\C,-1.3383677939,-1.082139533,0.0152495518\C,-1.34309
4774,0.3085136839,0.0113252572\C,-0.1657330535,1.059135553,-0.00510546
2\N,2.2935241053,0.9914694468,-0.0347429039\H,1.9823996873,-1.60983180
23,-0.023521028\H,-0.1627407787,-2.9169313,0.0062422737\O,-2.597424718
5,-1.544565602,0.0318461021\C,-2.7446175009,0.6450614283,0.0273623672\
H,-0.2835933271,2.1553397305,-0.0068143832\N,-3.477611207,-0.424417322
,0.0393157635\O,2.3024294739,2.2080242444,-0.038334093\O,3.3118436603,
0.3040086523,-0.0451269857\H,-3.0996572687,1.6839696711,0.0286945171\O
,-1.9299646566,3.2084164338,0.0101412571\H,-1.9766627645,4.1694819079,
0.0079560484\\Version=ES64L-G09RevE.01\State=1-A'\HF=-679.7884986\RMSD
=8.107e-09\RMSF=2.213e-05\Dipole=1.520389,-3.166252,-0.0093771\Quadrup
ole=-14.4388624,-1.324623,15.7634854,0.9722483,0.3636304,0.037145\PG=C
S [SG(C7H5N2O4)]\\@

TS

1\1\GINC-R3357\FTS\RM062X\6-31+G(d,p)\C7H5N2O4(1-)\ROOT\10-Oct-2016\0\
\# m062x/6-31+G** opt=(ts,calcfc,noeigen,maxcyc=200) scf=maxcyc=200 fr

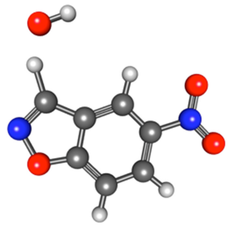

eq=noraman int=ultrafine\\TS for OH- H abstraction of oxazole\\-1,1\C,
1.0372351362,0.2687092505,-0.0164313151\C,1.0360883039,-1.1416851105,-
0.0124864605\C,-0.1547921698,-1.8469939494,0.003170938\C,-1.3291963788
,-1.0824708147,0.0145746685\C,-1.3096241798,0.311020016,0.0104964631\C
,-0.1219732349,1.0372410482,-0.0052245968\N,2.3371824014,0.9402796318,
-0.0332762016\H,1.9878988746,-1.6583132733,-0.0219990138\H,-0.17531597
28,-2.9306208041,0.0064014901\O,-2.5896174731,-1.5203899658,0.03028633
88\C,-2.7039141559,0.6948137301,0.0255020413\H,-0.1756223514,2.1275652
02,-0.0076707269\N,-3.458873025,-0.3557634376,0.037079686\O,2.35945033
14,2.1582514833,-0.0373368738\O,3.3482703821,0.2433444639,-0.042565709
4\H,-2.9249029408,1.836528488,0.0247777283\O,-2.1669113413,3.189624704
4,0.0119496155\H,-2.5421322061,4.075479337,0.0127519281\\Version=ES64L
-G09RevE.01\State=1-A\HF=-679.7875278\RMSD=5.536e-09\RMSF=2.145e-06\Di
pole=1.6871305,-2.866437,-0.01174\Quadrupole=-16.0716726,0.1590456,15.
912627,0.1013505,0.3690314,0.0407283\PG=C01 [X(C7H5N2O4)]\\@

product

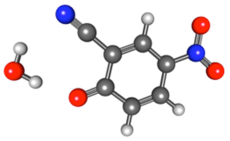

1\1\GINC-R46\FOpt\RM062X\6-31+G(d,p)\C7H5N2O4(1-)\ROOT\11-Oct-2016\0\\
m062x/6-31+G** opt=(maxcyc=200) scf=maxcyc=200 freq=noraman int=ultr
afine\\Product + H2O\\-1,1\C,1.6789970776,-0.5938672633,-0.0186338377\
C,0.8996792195,-1.7752429899,-0.0030240397\C,-0.4643487973,-1.70040068
43,0.0129941653\C,-1.1787209936,-0.4359763353,0.0148697938\C,-0.318086
11,0.7448786849,-0.0015421846\C,1.0650754819,0.657048106,-0.0177865719
\N,3.1037292758,-0.6805985372,-0.0353997711\H,1.4117540816,-2.73116364
11,-0.0041315123\H,-1.0752787283,-2.5971243549,0.0250806766\O,-2.42370
90638,-0.3708890589,0.0293484953\C,-0.9501093184,2.028039119,-0.000474
1293\H,1.6784240329,1.5511661208,-0.0298953672\N,-1.4681586072,3.06727
70895,0.0005203261\O,3.7637328767,0.3597816465,-0.0488648941\O,3.62533
08865,-1.7974332975,-0.0358252823\H,-3.7492950235,2.4905650059,0.03099
44486\O,-4.3666246354,1.7499568734,0.0420412346\H,-3.778587655,0.97324
25764,0.0388624498\\Version=ES64L-G09RevE.01\State=1-A\HF=-679.9133924
\RMSD=4.618e-09\RMSF=5.819e-06\Dipole=1.6671917,-1.0838485,-0.0143003\
Quadrupole=-32.8009999,10.8000063,22.0009936,14.7131016,0.5832667,-0.1
19791\PG=C01 [X(C7H5N2O4)]\\@
