# Supplementary material for: The evolution of multiple active site configurations in a designed enzyme
Source: Nat Commun. 2018 Sep 25;9:3900. doi: 10.1038/s41467-018-06305-y (PMC6156567; doi:10.1038/s41467-018-06305-y)
Supplement: Supplementary file 2 — Description of Additional Supplementary Files [file 41467_2018_6305_MOESM2_ESM.pdf]

Description of Additional Supplementary Files

File Name: Supplementary Data 1
Description: Data collection and refinement statistics
